# Ovarian cancer stem-like cells with induced translineage-differentiation capacity and are suppressed by alkaline phosphatase inhibitor

**DOI:** 10.18632/oncotarget.1424

**Published:** 2013-11-16

**Authors:** Kuei-Chun Liu, Yi-Te Yo, Rui-Lan Huang, Yu-Chi Wang, Yu-Ping Liao, Tien-Shuo Huang, Tai-Kuang Chao, Chi-Kang Lin, Shao-Ju Weng, Kuo-Hsing Ma, Cheng-Chang Chang, Mu-Hsien Yu, Hung-Cheng Lai

**Affiliations:** ^1^ Graduate Institute of Life Sciences, National Defense Medical Center, Taipei, Taiwan; ^2^ Department of Obstetrics and Gynecology, Tri-Service General Hospital, National Defense Medical Center, Taipei, Taiwan; ^3^ Department of Obstetrics and Gynecology, Shuang Ho Hospital, Taipei Medical University, Taipei, Taiwan; ^4^ Department of Pathology, Tri-Service General Hospital, National Defense Medical Center, Taipei, Taiwan; ^5^ Graduate Institute of Medical Sciences, National Defense Medical Center, Taipei, Taiwan; ^6^ Department of Biology and Anatomy, National Defense Medical Center, Taipei, Taiwan; ^7^ Graduate Institute of Biochemistry, National Defense Medical Center, Taipei, Taiwan; ^8^ School of Medicine, Taipei Medical University, Taipei, Taiwan

**Keywords:** alkaline phosphatase, cancer stem-like cells, epithelial-mesenchymal transition, epithelial ovarian cancer, levamisole, trans-lineage differentiation

## Abstract

Spheroid formation is one property of stem cells—such as embryo-derived or neural stem cells—that has been used for the enrichment of cancer stem-like cells (CSLCs). However, it is unclear whether CSLC-derived spheroids are heterogeneous or whether they share common embryonic stemness properties. Understanding these features might lead to novel therapeutic approaches. Ovarian carcinoma is a deadly disease of women. We identified two types of spheroids (SR1 and SR2) from ovarian cancer cell lines and patients' specimens according to their morphology. Both types expressed stemness markers and could self-renew and initiate tumors when a low number of cells were used. Only SR1 could differentiate into multiple-lineage cell types under specific induction conditions. SR1 spheroids could differentiate to SR2 spheroids through epithelial–mesenchymal transition. Alkaline phosphatase (ALP) was highly expressed in SR1 spheroids, decreased in SR2 spheroids, and was absent in differentiated progenies in accordance with the loss of stemness properties. We verified that ALP can be a marker for ovarian CSLCs, and patients with greater ALP expression is related to advanced clinical stages and have a higher risk of recurrence and lower survival rate. The ALP inhibitor, levamisole, disrupted the self-renewal of ovarian CSLCs in vitro and tumor growth in vivo. In summary, this research provides a plastic ovarian cancer stem cell model and a new understanding of the cross-link between stem cells and cancers. This results show that ovarian CSLCs can be suppressed by levamisole. Our findings demonstrated that some ovarian CSLCs may restore ALP activity, and this suggests that inhibition of ALP activity may present a new opportunity for treatment of ovarian cancer.

## INTRODUCTION

Differentiated descendant tissue cells do not exhibit some capacities of stem cells, such as being viable in suspension culture, and spheroid formation [1]. Stem cells can form spheroids in suspension, indicating that they are capable of proliferation, self-renewal and multipotency [1]. This property “spheroid formation” is usually applied to cancer stem-like cells (CSLCs) or spheroids for enrichment and to prove the stemness properties in those isolated cells [2, 3]. Such spheroid populations are generally ball-shaped, morula-like or irregularly shaped. It is not clear whether variations in morphology represent heterogeneous characteristics, such as differences in the stemness properties and hierarchical order, or whether they arise from natural properties in different types of cells. Distinct stemness markers or factors have also been proposed between the cancer stem-like spheroids from different types of tissues [3, 4]. The formation of cancer stem-like spheroids described in previous studies *in vitro* depended on special cell culture conditions rather than on the presence of spheroids forming during cancer development. Indeed, spheroid formation is a unique common phenomenon in ascites fluid of patients with ovarian cancers. It is still unclear whether spheroids formed from ovarian cancer are a single kind of CSLC [5-8]. To date, no study has investigated spheroids with diverse morphologies derived from ovarian cancers, especially those harvested from ascites or tumor tissues.

Our group previously characterized tumor-initiating spheroids expressing the surface markers CD44 and CD117 (c-Kit) [9]. Others have also reported that surface markers of ovarian tumor-initiating cells or ovarian CSLCs include CD133, CD24 and CD44/MyD88 [10-12]. Surface-marker-free methods also revealed side populations and quiescent CSLCs from ovarian cancer cell lines and other human cancerous tissues [7, 13-15]. The existence of ovarian epithelial stem cells is controversial, and there is insufficient evidence of the location of ovarian CSLCs within the abdominal cavity. A recent report has provided clues about the location of the stem cell niche for ovarian surface epithelial cells (OSEs) at the transitional area between the ovarian surface epithelium, mesothelium and tubal epithelium [16]. These OSEs from the hilum form spheroids in culture, show a dormancy-like phenotype, and display stem cell markers and long-term stem cell properties [16]. These stem-like and cancer-prone OSEs are thought to be the origin of high-grade serous type ovarian cancers. However, whether these stem-like OSEs possess translineage differentiation capability is not known, and whether the induced cancer cells retain stem-like properties has not been examined. There are insufficient data so far showing the direct transition of normal stem-like OSEs to ovarian CSLCs. In addition, the hierarchy of differentiation-related markers in CSLCs from melanomas and from colon and prostate cancers supports the stem-like origin of these cells [17-20]. However, the translineage differentiation capacity seen in pluripotent stem cells has not been observed in CSLCs [18-20]. The investigation of ovarian cancer stem-like spheroids might further elucidate the concept of cancer stemness in ovarian cancer development. Improving our understanding of ovarian CSLCs might also help in finding practicable therapeutic targets [15, 21, 22].

In the current study, we hypothesized that ovarian CSLCs would prove to possess different stemness status. We observed two types of ovarian CSLCs. Both of them fulfilled the definition of a CSLC, but only one of them possessed translineage differentiation capability. We also found that ovarian cancer development involves an epithelial–mesenchymal transition (EMT) in ovarian CSLCs. The suppression of alkaline phosphatase (ALP) activity inhibited the self-renewal and tumorigenicity of ovarian CSLCs. These findings provide evidence of a stemness hierarchy in ovarian cancer development. We also suggest that ALP might be a therapeutic target for women with ovarian cancers.

## RESULTS

### Two types of ovarian cancer spheroids have different morphologies and stem properties

**Figure 1:**
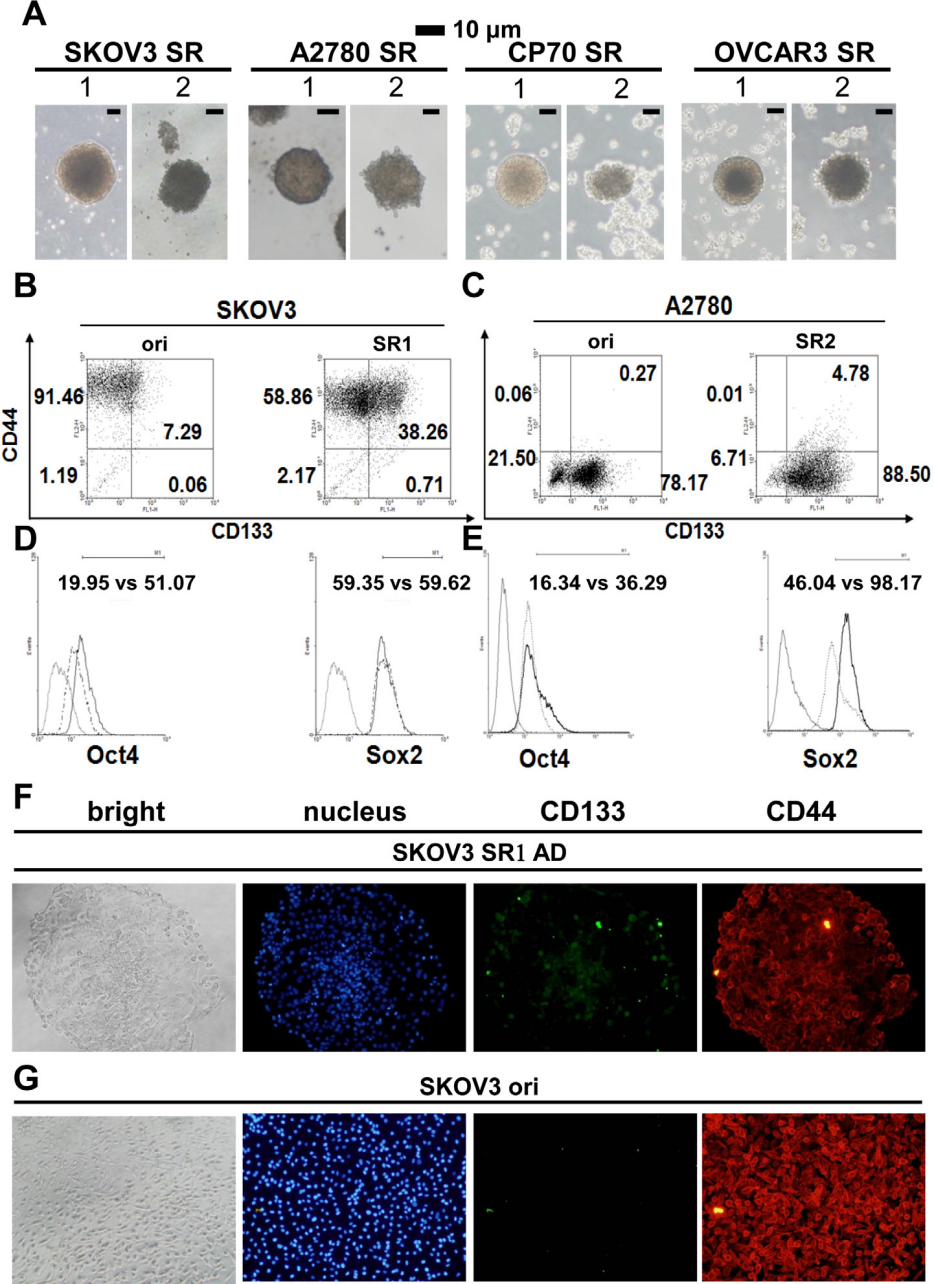
Morphology and stemness properties of ovarian cancer cell spheroids (A) Two different types of spheroids (SR1 and SR2) were enriched in various ovarian cancer cell lines. (B, C) The CD44^+^CD133^+^ signal increased in both SR1 and SR2 cells derived from SKOV3 and A2780 cells, whereas CD44^+^CD133^−^ and CD44^−^CD133^+^ were expressed in the original SKOV3 and A2780 cells, respectively. The expression of CD133 and OCT4 was higher in SKOV3SR1 cells (B, D), whereas the expression of OCT4 and SOX2 was higher in A2780SR2 cells (C, E). (F, G) Expression of CD133 and CD44 was compared between adhesive SKOV3-derived SR1 and the original cells. (F) After adhesive culture for 24 hours, the expression of CD133 decreased in adhesive SKOV3-derived SR1 from the center site to the periphery, but CD133 in scattered SR1 cells was still detectable in the periphery. (G) Almost all SKOV3 original cells expressed CD44 but not CD133.

We cultivated four human epithelial ovarian cancer (EOC) cell lines using stem cell suspended-culture conditions [23], which produced two distinctive types of spheres (SR1 and SR2) (Figure 1A). Each cell line preferred to develop into different types of spheres; the EOC cell lines SKOV3 and OVCAR-3 generated more SR1, A2780 cells formed more SR2, and CP70 cells could develop into both SR1 and SR2 simultaneously. SR1 exhibited a ball shape with a smooth surface, and SR2 was irregular in shape with a morula-like surface (Figure 1A). The morphology of SR1 and SR2 also differed under adhesion culture conditions (Supplementary Information, Figure S1A), For the assessment of stemness, flow cytometry was used to detect stem-associated markers in the two types of spheroids; the CD44^+^CD133^+^ population increased significantly in SKOV3SR1 and A2780SR2 cells, and the CD44^−^CD133^+^ population also increased in A2780SR2 cells (Figure 1B and 1C). Expression of OCT4 and SOX2 increased in SKOV3SR1 and A2780SR2 cells but with different magnitude of the change (Figure 1D and 1E). Immunocytochemistry staining confirmed that CD133 expression (Figure 1F and 1G) decreased significantly after induction of SKOV3SR1 differentiation.

### Single cell-derived type 1 spheroid clones express stemness markers and can differentiate into diverse morphologies

**Figure 2:**
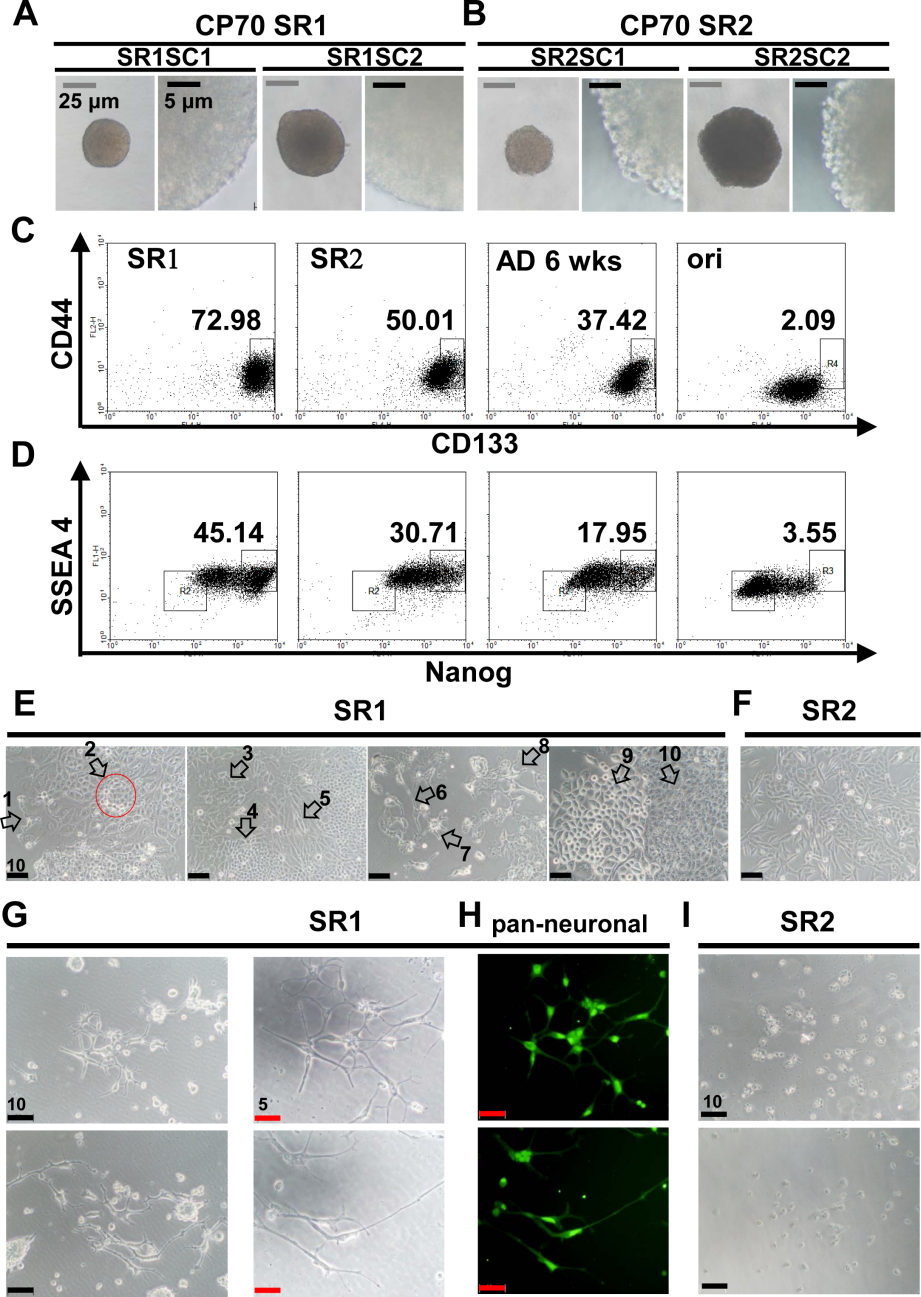
Characterization of single-cell clones isolated from CP70 SR1 and SR2 spheroids (A, B) Single cell-derived SR1 and SR2 clones are referred to as SR1SC and SR2SC, respectively. The surface of SR1SC (A) was smooth regardless of the size, whereas SR2SC (B) was morula like. (C, D) Stemness-associated markers were detected in single cell-derived SR1, SR2, and SR cells 6 weeks after differentiation, and in the original cells. (C) The population of CD133^+^/CD44^+^ cells was the highest in SR1 followed by SR2, differentiated SR cells, and the original cells. (D) Expression of both the stemness-associated marker NANOG and hESC-related marker SSEA4 was increased in the order SR1, SR2, and SR differentiated cells. SSEA4 expression was lower in CP70 original cells than in their counterpart SR cells. (E) Dissociated SR1SC cells were induced to differentiate for 14 days. At least 10 types of morphology (white arrows) with a neuron-like phenotype were observed in different fields of view, whereas CP70SR2 cells differentiated only to a simple and tumor-like morphology (F). Neuron-like morphology was observed only in CP70SR1 cells (G), and expression of multiple neuronal markers was detected by immunostaining using anti-pan-neuronal antibodies (H). However, most CP70SR2 cells were dead when treated under the condition described above for 16 days (I).

A diversity of morphology was observed in the suspension cultures of original CP70 cells (Supplementary Information, Figure S1B). To assess stemness properties of single cell-derived CSLC, pure populations of CP70SR1 and SR2 were procured (Supplementary Information, Figure S1C) [18, 24]. CP70SR1 and SR2 clones derived from single cells (also known as CP70SR1SC and CP70SR2SC, respectively) were isolated by limiting dilution. The size of the spheres did not appear to affect the distinctive appearance (Figure 2A and 2B); however, self-renewal was slower for SR1 than for SR2, suggesting SR1 was more dormant than SR2 [13]. Flow cytometry was used to identify the expression of CD44 [9, 21], CD117 [9], CD133 [6, 11], NANOG, and SSEA4 [25]. There was an order of stem-like cell hierarchy between CP70SR1, SR2, differentiated SR, and original CP70 cells (Figure 2C and 2D, Supplementary Information, Figure S1D). Interestingly, CP70SR1SC branched out and exhibited at least 10 different morphologies (Figure 2E). By contrast, CP70SR2SC showed only basic tumor cell morphology (Figure 2F).

To determine whether SR1 and SR2 retained their tumorigenic capacity as in ovarian CSLCs [9, 11, 13], we tested this using an *in vivo* animal model of tumorigenesis. As few as 1000 CP70SR1SC cells could propagate a visible tumor nodule 6 weeks after implantation (Supplementary Information, Figures S2A-C). The tumor sections were classified as a poorly differentiated malignancy of human origin (Supplementary Information, Figure S2D). The high tumorigenicity of CP70SR2SC was also confirmed *in vivo* (Supplementary Information, Figure S3). Various markers were applied in immunohistochemistry to determine whether CP70SR1SC could differentiate spontaneously and propagate tumors *in vivo*. The expression of CD44 and CD133 decreased (Supplementary Information, Figure S2E) and, to our surprise, human-specific endothelial-associated marker CD34 increased (Supplementary Information, Figure S2F) in the tumor nodules. These results suggest that both SR1 and SR2 fulfill the definition of CSLCs requirement.

### Translineage differentiation capability of type 1 ovarian cancer stem-like spheroids

The differentiated CP70SR1SC exhibited dendrite- and axon-like morphology (Figure 2E), and various conditions were applied [26-28] to assess the neurogenesis potency. Single cells dissociated from CP70SR1SC (Supplementary Information, Figure S4A) under induction exhibited neuron-like morphology and expressed the premature neural marker α-internexin, and this could be further demonstrated by the observation of induced an entire CP70SR1SC neuronal-like differentiation (Supplementary Information, Figure S4D). This phenomenon was present only in induced SR1 (Supplementary Information, Figure S4A and S4B) and not in SR2 (Supplementary Information, Figure S4C). Long-term treatment of CP70SR1 under the modified induction condition produced more neuron-like morphology (Figure 2G), and the cells expressed at least one of four types of neural-associated markers (Figure 2H, Supplementary Information, Figure S4E). However, no CP70SR2 survived in the same condition (Figure 2I). Furthermore, in the same condition, SKOV3SR1 cells also exhibited elongate dendrite- and axon-like morphology and contained small lipid droplets [29, 30] (Supplementary Information, Figure S4F).

**Figure 3:**
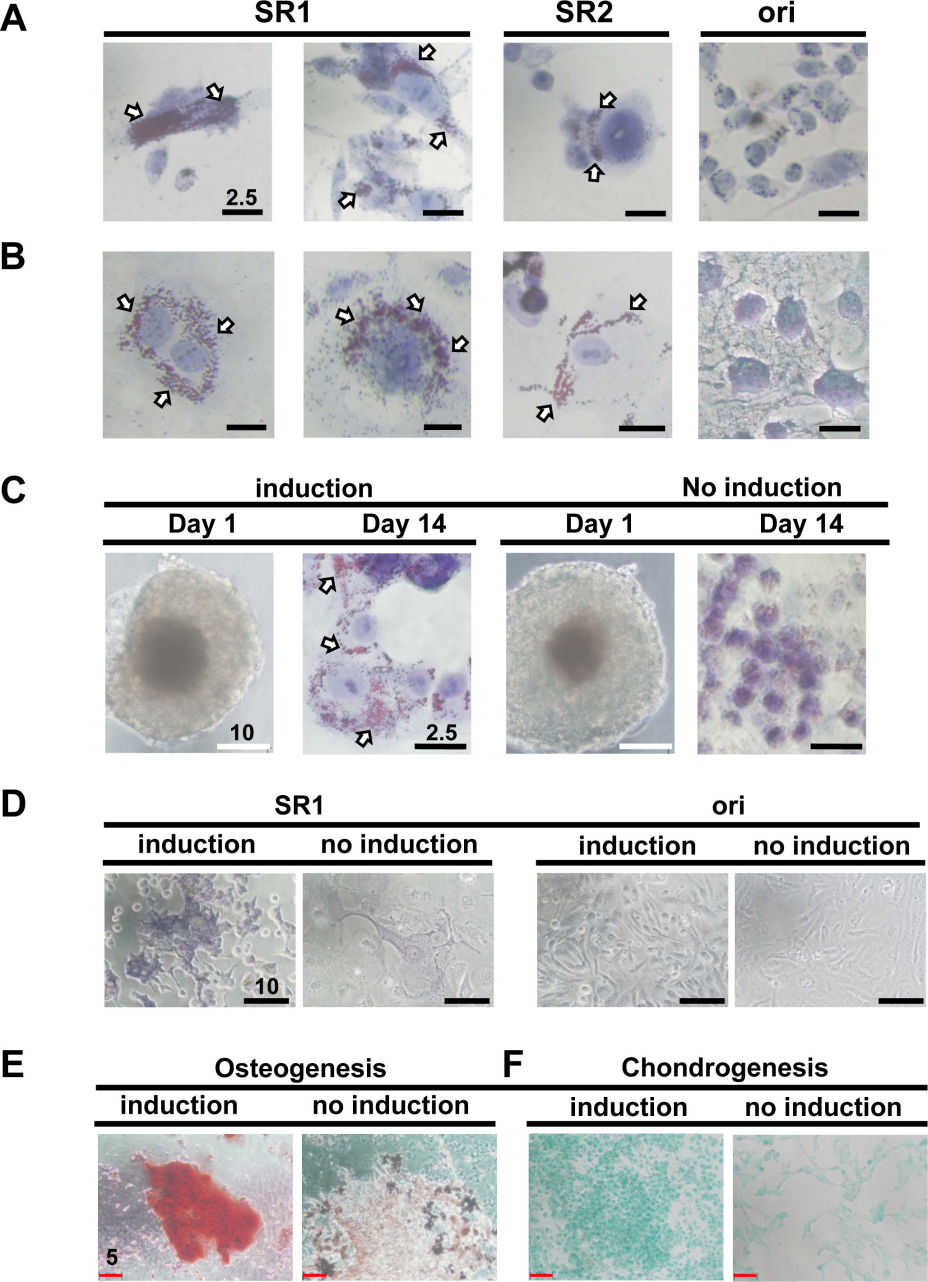
SR1 cells derived from various ovarian cancer cell lines show multiple differentiation potency (A, B) SR1, SR2, and their original counterpart cells from CP70 (A) and SKOV3 (B) cells were induced by adipogenesis-inducing medium for 14 days and stained with oil red O solution. Only SR1 and SR2 cells, but not the original cells, displayed lipid droplets in the cell body (white arrows). (C) Two independent clones of SR1 cells derived from OVCAR3 cells were seeded directly onto collagen-coating culture dishes in either adipogenesis-inducing medium or SR culture medium. Oil droplets were observed only in SR1 cells cultured in adipogenesis-inducing medium. (D) The osteogenesis capacity of SKOV3-derived SR1 cells and its original counterpart cells were examined. Only SR1 cells cultured in osteogenesis-inducing medium displayed osteocyte morphology with the osteogenic marker, ALP activity, 14 days after induction. Differentiated CP70SR1 were induced by osteogenesis (E) and chondrogenesis (F) and detected by specific staining. Undifferentiated CP70SR1 without induction retained a tumor-like morphology and showed no signals.

After observing this change in morphology (Figure 2E), we confirmed the adipogenic potency of CP70- and SKOV3-derived SR1 and SR2 (Figure 3A and 3B). The original tumor cells barely exhibited this differentiated capacity (Figure 3A and 3B). Only OVCAR-3SR1 differentiated into adipocyte-like cells under the induction condition (Figure 3C). Specific dye staining demonstrated the osteogenic induction of SKOV-3SR1 and CP70SR1 (Figure 3D and 3E) as well as chondrogenesis of CP70SR1 (Figure 3F).

**Figure 4:**
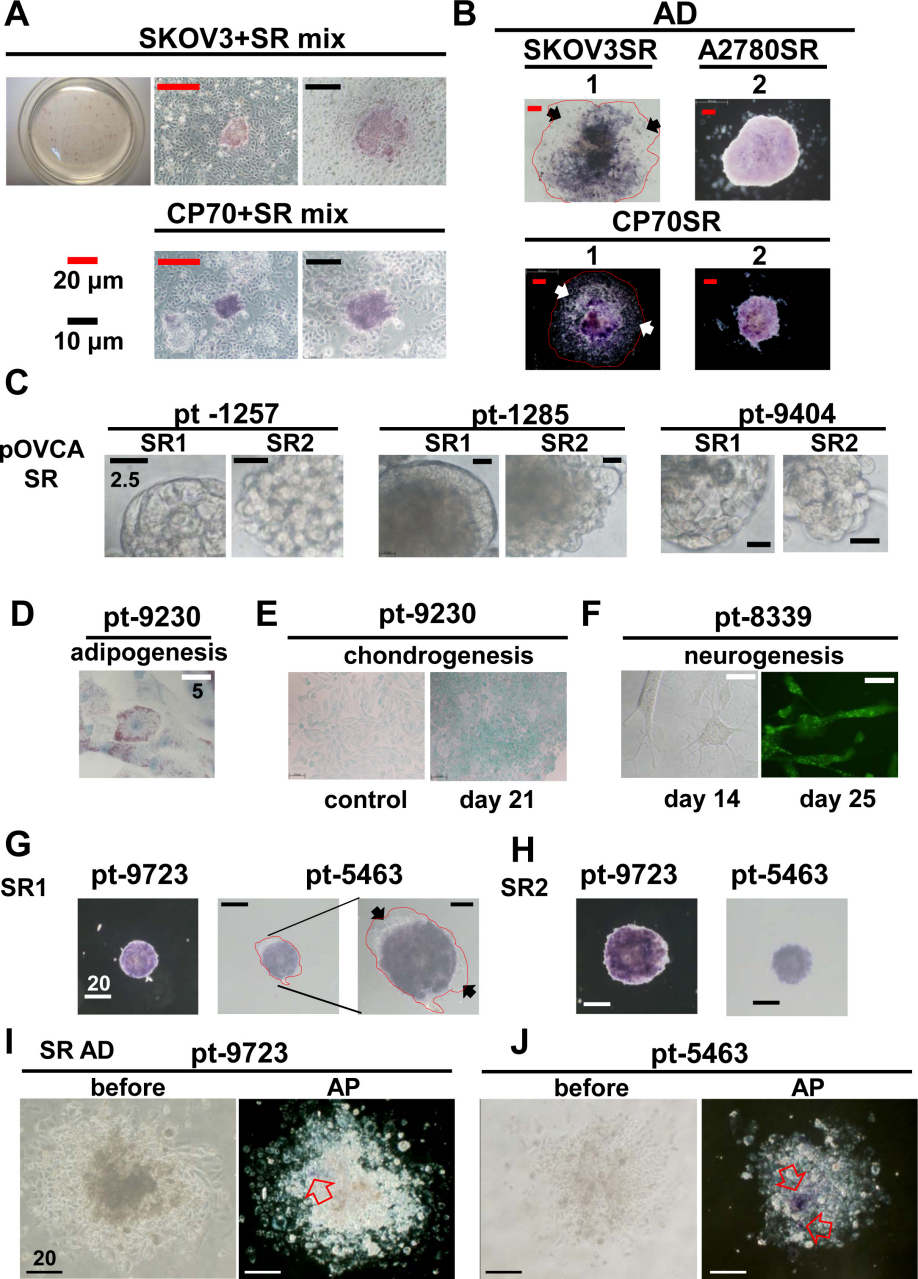
Stemness-associated marker ALP increased in spheroid cells derived from ovarian cancer cell lines and patients' tissues (A) ALP activity was assessed in spheroids and aggregated counterpart human SKOV3 and CP70 cells 18 h after attachment to the culture plate. ALP activity was detected only in spheroids in both cell lines. (B) ALP activity was upregulated in spheroids from three different cell lines, compared with their differentiated forms in cells that were allowed to attach to the culture plate in standard medium for 3–5 days. The red line encircles the extended region of adhesive SR1 cells, and arrows indicate ALP-negative differentiated SR1 cells. (C) SR1 and SR2 cell lines were isolated from ascites fluids from patients with ovarian cancers. Most spheroids displayed SR2 morphology. (D–F) SR cells isolated from patients' tissue were enriched and passaged three times for further assays. (D) After adipogenesis induction, SR cells could differentiate into adipocytes and displayed oil droplets in the cell bodies. (E) Alcian blue staining confirmed that isolated SR cells showed chondrogenic morphology and displayed the chondrocyte-specific marker 21 days after the induction of chondrogenesis. (F) Isolated SR cells exhibited a neuron-like morphology 14 days after induction and expressed α^−^internexin 21 days after induction. Patient specimens (solid tumor)-derived SR1 (G) and SR2 (H) cells were cultured in standard culture dishes for 6 h, and ALP activity was measured. (G) ALP activity was detectable only in undifferentiated SR cells and not in differentiated cells (black arrows). The red line encircles the extended region of adhesive SR1 cells. (I, J) When cultured in standard medium under adhesion conditions, after 7 days, ALP activity decreased significantly in differentiated SR cells derived from two individual patients. The left panel shows a bright field view of differentiated SR cells before ALP activity staining; the right panel shows the region of remaining ALP activity in the differentiated SR cells (open arrow).

Alkaline phosphatase is a hydrolase responsible for removing phosphate groups from nucleotides [31], proteins, and alkaloids. ALP activity can be used to test the stemness pluripotency of ESCs or embryonic carcinoma cells [32-34]. We tested whether ALP was active in ovarian cancer stem-like spheroids enriched from cell lines and patients' specimens. However, only some spheroids possessed distinct ALP activity (Figure 4A). For confirmation of ALP activity, a single SR1 spheroid was dissected, and almost every cell was ALP positive (Supplementary Information, Figure S5A and S5B); however, the ALP activity decreased in SR progenies after a prolonged period of differentiation (Figure 4B). Both types of spheres were observed in ascites harvested from different EOC patients without culturing (Figure 4C). SR1 and SR2 could also be enriched from cancer tissues after culture. In clinical specimen-derived SR1, the capacities for adipogenesis (Figure 4D), chondrogenesis (Figure 4E), and neurogenesis (Figure 4F) were confirmed, further supporting the potency of the translineage differentiation. Furthermore, the ALP activity (Figure 4G and 4H) decreased or was lost after differentiation in SR progenies derived from ascites and cancer tissues (Figure 4I and 4J, Supplementary Information, Figure S5C).

### Differentiation of type 1 spheroids to cancer progenies is accompanied by EMT and loss of ALP expression

A hierarchy of stemness could be defined between SR1, SR2, and their differentiated progenies (Figure 2C and 2D, Supplementary Information, Figure S1D). EMT was reported to be involved in the stemness of cancer cells [35-37]. However, the EMT status of SR1 and SR2 is unknown. During the modified culture, some single SKOV3- (Supplementary Information, Figure S5D) and CP70-derived SR1 (Supplementary Information, Figure S5E) showed the simultaneous appearance of features of both SR1 and SR2.

**Figure 5:**
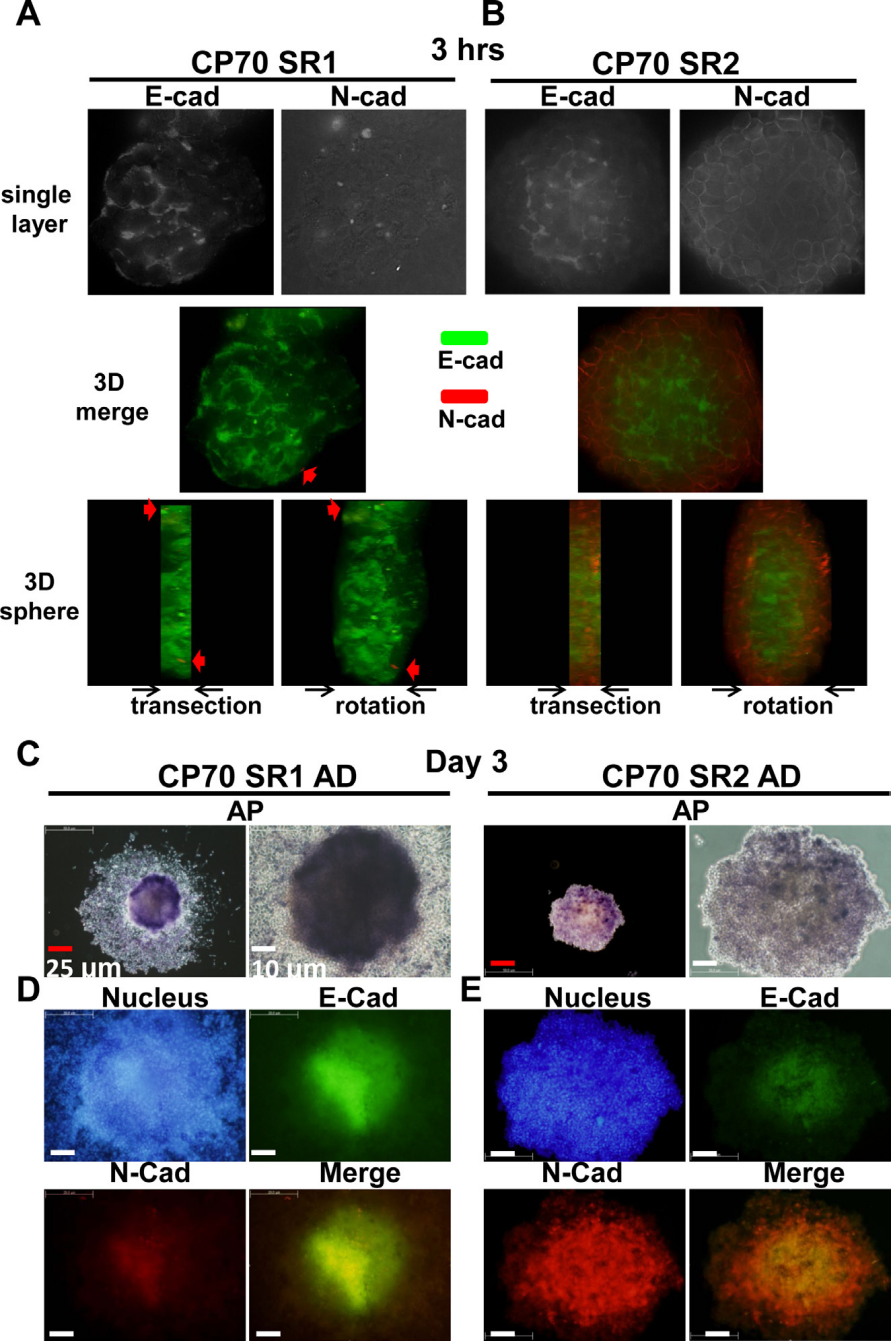
Differences in ALP activity and expression of E-cadherin (E-cad) and N-cadherin (N-cad) between SR1 and SR2 cells Entire spheroids of CP70SR1 (A) and CP70SR2 (B) were seeded into culture dishes for 3 hours and stained with combined antibodies against E-cad and N-cad (red arrow), and protein expression was analyzed by three-dimensional dissection using the DeltaVision system. E-cadherin was expressed throughout the entire SR1, only in the center of SR2, and N-cad was expressed only at the border. (C) After differentiation, SR1 cells grown under the adhesion condition showed a different pattern of ALP activity from the center to the periphery. SR2 cells showed weaker adhesion ability, and ALP activity was detectable only in certain regions. (D, E) The expression and location of E-cad and N-cad differed significantly between SR1 and SR2. The expression of E-cad and N-cad decreased from undifferentiated cells (center) to differentiated cells (boundary) in both SR1 and SR2. (D) E-cadherin was expressed mainly in the center of adhesive SR1, whereas the expression of N-cad was weaker at the same site. (E) In adhesive SR2, N-cad was expressed in most cells but with different strength, whereas E-cad was detectable only in the center.

We next investigated the roles of EMT in SR1 and SR2 using the expression of E-cadherin and N-cadherin as an index [38, 39]. Increasing evidence indicates that E-cadherin expression maintains colony formation and self-renewal of hESCs [40, 41] and neural stem cells [42], and increases the generation of induced pluripotent stem cells [43, 44]. In addition, declining E-cadherin expression will result in the loss of multipotency [42]. E-cadherin expression was found in most of the SR1 main body, while N-cadherin was only detected in a few spots (Figure 5A). Interestingly, the entire SR2 showed an EMT-like situation; E-cadherin was expressed dominantly in the center of the spheroid and was surrounded by the N-cadherin signal in the periphery (Figure 5B). After long-term differentiation, ALP activity was lost in the periphery of the differentiated SR1 and SR2 (Figure 5C). E-cadherin was expressed mainly in the center of SR1, similar to the ALP signal (Figure 5D), where the expression of N-cadherin was weaker (Figure 5D). The loss of ALP activity and EMT occurring in differentiated SR1 is similar to the differentiated process of human ESCs (hESCs) [34, 45]. Differentiated SR2 maintained high expression of N-cadherin in the center, but the expression decreased in the periphery (Figure 5E). ALP activity (Supplementary Information, Figure S6A) and the distribution of E-cadherin and N-cadherin (Supplementary Information, Figure S6B) also differed significantly between SR1 and SR2. These results indicated SR1 that stays at the higher order of stem-like cell hierarchy showed a more epithelial-like appearance, and that SR2 may originate from SR1.

### ALP as a potential therapeutic target and poor prognostic factor for patients with ovarian cancers

**Figure 6:**
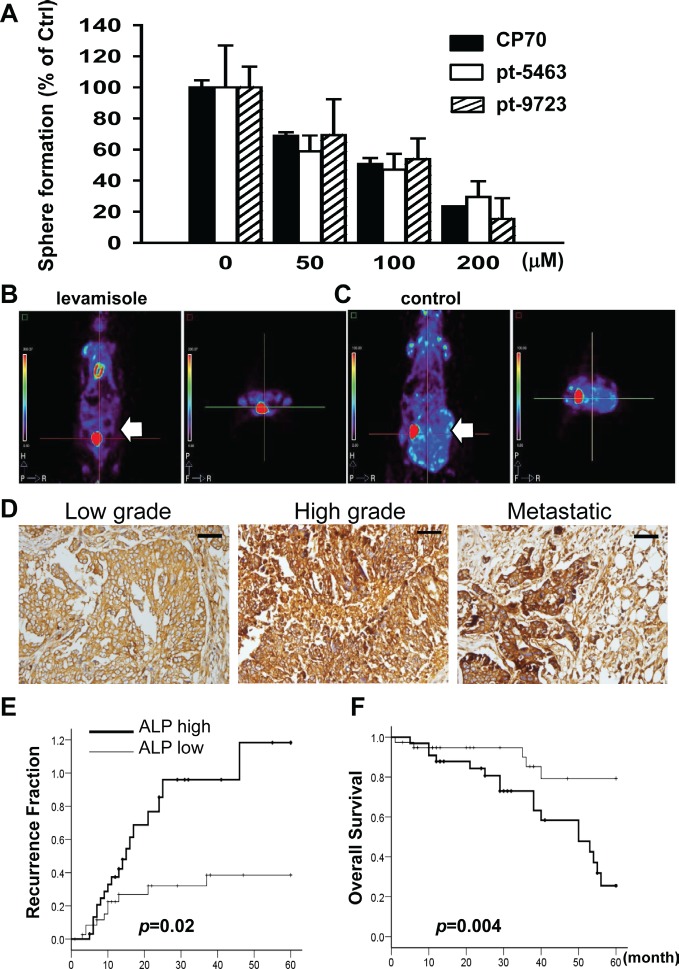
Inhibition of ALP activity by levamisole interferes with the formation of spheroids and tumors in ovarian CSLCs (A) Levamisole inhibited spheroid formation of dissociated single CP70SR1 cells and specimen-derived SR1 cells. The inhibitory efficacy of levamisole differed between spheroids isolated from different patients. For *in vivo* analysis of the effect of levamisole on ovarian CSLCs, mice were implanted peritoneally with CP70 cells and then given oral levamisole (B) or placebo (C). Tumor volume was significantly decreased in levamisole-treated mice as analyzed using a micro-PET image system. (D) ALP expression was associated with serous type and a high grade of EOC. In addition, metastatic cancer with the greatest expression of ALP indicated that ALP was related with advanced stage of EOC. Kaplan–Meier analysis of the probability of recurrence (E) and overall survival rate (F) in ovarian cancer patients stratified according to ALP expression (n = 73).

The upregulation of ALP in hepatocellular CSLCs has been reported recently [25]. We aimed to clarify whether ALP plays an important role in ovarian CSLCs. Levamisole suppressed sphere formation of SR1 originating from the CP70 cell line and human specimens (Figure 6A). The quantity of spheres formed and basic morphology of these spheres were also significantly affected, indicating that ALP inactivation inhibited ovarian CSLC self-renewal (Supplementary Information, Figure S6C). Tumor formation was repressed only in mice that had received oral levamisole (Figure 6B and 6C).

The role of ALP in human EOC was verified by immunohistochemical staining using ovarian cancer tissue arrays [21, 46]. ALP expression was higher in malignant tumors (Figure 6D, Supplementary Information, Figure S7A and S7B) than in benign and borderline tumors (Supplementary Information, Figure S7A-C). EOC patients were classified according to ALP expression scores into two groups: ALP high (score>170) and ALP low (score=0–170) (Supplementary Information, Figure S7A-C, Table 1). Expression of ALP was significantly associated with histological type (*p*=.002) and pathogenesis model [47] (*P*<0.001) (Supplementary Information, Figure S7A, S7B, and S7D, Table 2). Patients with greater ALP expression exhibited a higher recurrence ratio (Figure 6E and 6F) (*p*=.02) and lower overall survival (*p*=.004). Multivariate analysis revealed that ALP expression was also strongly associated with cancer stage and nuclear grade, indicating ALP was not an independent factor in the recurrence and survival in patients (Table 2). To sum up, the existence of ALP in cancer cells may be associated with stem properties and tumorigenesis of EOC cells.

**Table 1:** Clinicopathological features

	ALP score				
	0–170		>170		p value ^a^
Patients, n	40		33		
Age (years)					0.43
Range	16-79		37-76		
Mean ± SEM	51.3 ± 2.6		53.9 ± 1.9		
Stage, n (%)					**<0.001**
I, II	24	(82.8)	5	(17.2)	
III, IV	16	(36.4)	28	(63.6)	
Nuclear grade, n (%)					**0.009**
Low (G1, G2)	24	(72.7)	9	(27.3)	
High (G3)	16	(40.0)	24	(60.0)	
Histological type, n (%)					**0.002**
Serous type	21	(42.0)	29	(58.0)	
Other types	19	(82.6)	4	(17.4)	
Pathogenesis model, n (%)					**<0.001**
Type I	23	(95.8)	1	(4.2)	
Type II	17	(34.7)	32	(65.3)	

a Significantly in bold (p < 0.05)

**Table 2:** Multivariate analysis of clinicopathological factors in 73 ovarian cancer patients ^a^

	Recurrence		Survival	
Variable	Univariate analysis crude HR (95% CI)	Multivariate adjusted HR (95% CI) ^b^	Univariate analysis crude HR (95% CI)	Multivariate adjusted HR (95% CI) ^e^
Age (years)	1.03 (1.00–1.06)	-	1.05 (1.02–1.09) ^d^	1.03 (0.99–1.07)
ALP expression ^c^				
Low	1.00 (reference)	1.00 (reference)	1.00 (reference)	1.00 (reference)
High	2.41 (1.12–5.21) ^d^	0.88 (0.39–2.01)	3.91 (1.44–10.65) ^d^	1.00 (0.33–3.00)
Stage				
I, II	1.00 (reference)	1.00 (reference)	1.00 (reference)	1.00 (reference)
III, IV	16.07 (3.77–68.55) ^d^	25.99 (4.36–154.97) ^d^	19.94 (2.67–148.64) ^d^	14.35 (1.78–115.85) ^d^
Grade (nuclear)				
Low	1.00 (reference)	1.00 (reference)	1.00 (reference)	1.00 (reference)
High	4.32 (1.81–10.32) ^d^	3.22 (1.18–8.73) ^d^	5.83 (2.07–16.42) ^d^	3.44 (1.03–11.55) ^d^
Histological type				
Serous type	3.54 (1.07-11.74) ^d^	0.26 (0.05-1.32)	2.09 (0.62-7.07)	-
Other types	1.00 (reference)	1.00 (reference)	1.00 (reference)	-

Abbreviations: HR, hazard ratio; CI, confidence interval

a Cox proportional hazards model was applied

b The analysis adjusted for ALP expression, stage, nuclear grade and histological type

c The low expression of ALP regarding survival is represented as 0–170 and the high expression of ALP regarding survival is represented as > 170.

d Significantly correlated with outcome, p < 0.05

e The analysis adjusted for age, ALP expression, stage and nuclear grade

## DISCUSSION

The CSLC hypothesis proposes that tumors exhibit a hierarchical organization and that only a subset of cells with stem cell properties possess self-renewal capacity, drive tumor initiation and sustain tumor growth [4, 48]. Different models of carcinogenesis based on this concept have been proposed [9, 24, 49, 50]. Several groups have claimed to identify ovarian CSLCs [7, 9-14, 50] but have suggested that ovarian CSLCs are heterogeneous populations [51]. Our results demonstrate that using the current definitions, ovarian CSLCs are diverse in terms of spheroid morphology and stemness status. In this study, the varying expression levels of CD133, CD44, and ALP, and distinct phenotypes in ovarian CSLCs from different EOC cell lines and from the same cell populations reveal heterogeneity of the CSLC phenotype [51]. This implies that there must be functional and phenotypic diversity among these CSLCs [52]. In the CSLC model, most cancers might be monoclonal in origin; however, they can differentiate into all cell types in the tumor and then generate intratumor heterogeneity [48]. This phenotypic and genetic heterogeneity plays an important role in neoplasia, cancer progression, and resistance to therapies [51].

A recent report revealed two distinct spheroid types in glioma stem cells (GSCs), in which the mesenchymal type GSCs are more aggressive and tumorigenic than the proneural type [53]. Although these two types of GSCs possess mutually exclusive signaling pathways, radiation treatment of proneural GSCs upregulated mesenchymal markers and downregulated proneural markers, suggesting the possibility of a transition between different cancer stem cell populations. The present study demonstrated two types of cancer stem-like spheroids in human ovarian cancer cell lines and cancer tissues. The transition between different types of CSLCs spheroids suggests the important role of cancer stem plasticity in drug resistance, tumor recurrence and metastasis [51]. The microenvironmental cues triggering these transitions remain unknown. In addition, both glycolysis and aldehyde dehydrogenase (ALDH) activity were significantly elevated in mesenchymal GSCs but not in proneural GSCs, which makes targeting the ALDH signaling pathway a potential approach for glioma treatment. The present study demonstrated that ALP might serve as a potential therapeutic target for patients with ovarian cancers. However, any differences in metabolism between ovarian CSLCs and non-stem counterparts remain unexplored. Indeed, the function of ALP is associated with distinct networks for cell physiology [31, 54-56]. We have reported that niclosamide can disrupt multiple metabolic pathways in ovarian CSLCs to achieve tumor inhibition [15], supporting the potential of metabolic targeting in ovarian cancer treatment.

Translineage differentiation is one capacity of normal stem cells, and it determines the hierarchical status of cells. The stemness definition of CSLCs remains controversial [57]; however, they appear similar to normal stem cells, suggesting that some of the CSLCs might also differentiate into different cell lineages other than the original lineage from which the tumor arose. In the present study, we found that conditional induction could lead ovarian CSLCs to differentiate into distinct lineages of cells, although the effectors or mechanisms are unknown. However, this capability of CSLCs for translineage differentiation might offer a therapeutic strategy that has not yet been fully explored [22].

According to a recent study, there is a cancer-prone stem cell niche at the junctional area of the ovarian surface epithelium, and one population of normal cells bearing stem-like properties exists in this specific site [16]. This finding [16] implies the possible existence of ovarian CSLCs showing slow cycling and asymmetric propagation into differentiated cancer cells, which could be enriched from the ovarian cancer cell population. Although various signals in cancer stem cells could be distinct from normal stem cells, in our study, the SR1 spheroids exhibited multiple characteristics including a distinct morphology, expression of the stem-associated markers ALDH1 (data not shown) and CD133, and slow cycling. These were similar to the properties of OSE stem cells from the hilum of the ovary reported by Flesken-Nikitin et al.[16].

We speculate that the presence of cancer tissues with high ALP expression might imply that ovarian cancer cells have gained some stemness characteristics and that this might be related to the high rates of recurrence and death of patients with such tumors. As for stemness, the regulation of ALP expression is associated with β-catenin signaling [58] and epigenetic modulation [59, 60], and its enzymatic activity is inhibited by levamisole [61]. Levamisole was developed originally as an anthelmintic to treat parasitic infection in both humans and animals. In recent decades, the combination of 5-fluorouracil and adjuvant levamisole (adjuvant chemotherapy) had been the standard clinical protocol for treating patients with colon cancers. The therapeutic efficacy of adjuvant chemotherapy in cancer treatment is controversial [62-69]. Two independent studies of levamisole in ovarian cancer treatment have shown opposite results [62, 63]. We speculate that these differences could reflect different levamisole dosages, dosing intervals or patient selection in the different clinical trials. Applying newly designed chemotherapy drugs in combination with levamisole instead of an outmoded drug such as melphalan [62, 63] and a better design of patient selection in clinical trials might produce better efficacy in ovarian cancer treatment. We suspect that one of the tumor-inhibitory effects of levamisole might be through the suppression of ALP activity, because knockdown of ALP could impair stemness properties [70], and this might result in repression of tumor propagation. More evidence is needed to justify the clinical use levamisole and its derivatives for ovarian cancer treatment. We anticipate that inhibition of ALP activity might present a promising strategy against a subset of EOCs.

The association between EMT and stemness remains controversial. The induction of EMT promoted the growth of cancers exhibiting stemness properties [35-37]. However, others reported contrary results [42, 71-74]. Thus, EMT is associated with varied functional phenotypes in different cancers. The epithelial-like status marker E-cadherin is a key tool for analysis of the three-dimensional structure of embryoid bodies and neurospheroids [42, 71]. Indeed, optimal reprogramming of target cells for induced pluripotent stem cells is a sequential EMT−MET process [75]. In this study, E-cadherin was abundant in SR1 spheroids but decreased in the SR2 spheroids, which suggests an EMT process. It remains unclear whether differentiated EOC cells regain E-cadherin expression directly or progress to the MET to further acquire the stemness phenotype. Our results support the idea that EMT status is a plastic functional phenotype of ovarian cancers [51, 76].

In summary, this research supports a plastic ovarian cancer stem cell model and provides a new understanding of the cross-link between stem cells and cancers. ALP was confirmed as a potential therapeutic target for treating women with ovarian cancers. An immediate trial of a clinically proven ALP inhibitor may facilitate the translation of cancer stem cell research to patient therapy.

## MATERIALS AND METHODS

### Enrichment of Spheroid Cells and Establishment of Single Cell-Forming Spheroids

The original human ovarian cancer cell lines OVCAR-3 and SKOV3 were purchased from ATCC (Manassas, VA, http://www.atcc.org). The original human ovarian cancer cell line A2780 and its cognate cisplatin-resistant CP70 were obtained in 2007 from Dr. Tim Huang's lab (University of Texas Health Science Center, San Antonio, TX). The cell lines used were tested by the Bioresource Collection and Research Center (Hsinchu, Taiwan) for identity verification by DNA profiling of short tandem repeat sequences. DNA profiles were compared manually to the ATCC and European Collection of Cell Cultures database. Earlier passages of all cell lines were maintained in several cryovials in liquid nitrogen in our laboratory. All original cell lines were cultured in RPMI-1640 (Invitrogen) supplemented with nonessential amino acids (Invitrogen), sodium pyruvate (Invitrogen), and either 10%–20% standard fetal bovine serum (FBS, Biological Industries) or different growth factors, and plated in standard cell culture dishes (Corning). To enrich spheroid cells carrying stemness properties, the original cancer cells from cell lines or primary culture were harvested and cultured in ultra-low attachment plates (Corning). Following the culture protocols for ESCs and neuronal stem cells, cells were cultured in Dulbecco's modified Eagle medium (DMEM) with a high or low glucose concentration, or DMEM/F12 medium containing 0%–5% FBS qualified for use with human ESCs (Biological Industries) or KnockOut™ Serum Replacement (Invitrogen), supplied with or without basic fibroblast growth factor (bFGF; PeproTech), and either human recombinant epidermal growth factor (EGF; PeproTech) or 2-mercaptoethanol (Millipore), depending on the protocol modified for different ovarian cancer cell lines or patients' samples. The cells were cultured in suspension, and starting from 14 days, the cultures were examined every day for sphere formation. Spheres were then dissociated and passaged at least eight times in 2 months to generate spheres, which are henceforth referred to as “SR cells”. Collection of each clinical specimen adhered to the protocol approved by the institutional review board.

### Characterization of ALP Activity and Stem-Like Cell Properties of SR Cells

The ALP activity in parental cancer cells, SR cells, and differentiated SR cells was assayed using an Alkaline Phosphatase Detection Kit (Millipore). The stem-like phenotypes of the parental CP70 and SR cells were assessed by detecting specific stem cell gene markers using flow cytometry (BD Biosciences) and immunocytochemistry. Cells were stained intracellularly with antibodies against human NANOG (GeneTex), OCT4 (Millipore), stagespecific embryonic antigen 4 (SSEA4; BioLegend), and SOX2 (Millipore), according to the manufacturer's instructions as described previously [15, 21]. The cells were stained with CD44 (BioLegend), CD105 (BioLegend), CD117 (BioLegend), CD133 (Abcam), E-cadherin (BioLegend), or N-cadherin (BioLegend) fluorescence-conjugated monoclonal antibodies and analyzed by flow cytometry, fluorescence microscopy, or the DeltaVision Imaging System (DV Elite™ System; Applied Precision).

### Tumorigenic Capacity of SR Cells

For *in vivo* tumor xenograft studies of CSLCs, female nonobese diabetic/severe combined immunodeficient (NOD/SCID) mice were obtained from the Laboratory Animal Center of the National Taiwan University College of Medicine (Taipei City, Taiwan). Six-week-old mice were used for the experiments unless otherwise indicated. The rules of the Animal Protection Act of Taiwan were strictly followed, and all animal procedures were approved by the Laboratory Animal Care and Use Committee of the National Defense Medical Center. Various numbers of SR cells and original cancer cells were injected subcutaneously or intraperitoneally (i.p.) into NOD/SCID mice. Tumor vessels in the tumor nodule in live mice were quantified after 14 days by measuring the tumor uptake of the blood pool probe AngioSense™ 750 [77, 78] and analyzed using the VisEn FMT image system (PerkinElmer) [78]. Tumor formation was observed 10 days after injection. Mice were euthanized when the swollen abdomen (an indication of tumor formation) was observed. Tumor nodules were harvested, and the sections were analyzed by hematoxylin and eosin staining to confirm the origin of the tumor cells. Other specimens were analyzed by immunohistochemistry using specific antibodies against human blood vessels (CD34; Dako) and stemness-associated markers.

### Differentiation of Cancer Stem-Like Cells

For differentiation studies, SR cells were dissociated and cultured in standard medium containing 5% standard FBS in standard dishes (Corning) coated with FNC Coating Mix® (AthenaES). The morphology of spontaneously differentiated SR cells was observed, and the translineage-differentiating capacity was tested based on various protocols for inducing the differentiation of adipocytes, osteocytes, chondrocytes, and neural cells. SR cells were dissociated and allowed to form aggregates for 2 days, and neural differentiation was induced. The cells were transferred to poly-d-lysine-coated dishes and cultured in common medium containing retinoic acid for 4 days. Neural differentiation of SR cells was also induced using Neurobasal® (Invitrogen), DMEM/F12 (Invitrogen), or DMEM/F12 knockout (Invitrogen) medium supplied with B27 (Invitrogen), N2 (Invitrogen), human recombinant bFGF (PeproTech), and EGF (PeproTech). Neural cell differentiation was confirmed using immunostaining with antibodies against α-internexin (GeneTex) and βIII-tubulin (GeneTex), and pan-neuronal markers (NeuN, βIII-tubulin, MALP2, and NF-H; Millipore). Mesenchymal differentiation of SR cells was induced using a commercial StemPro® Differentiation Kit (Invitrogen). Adipocyte formation was confirmed using oil red O staining. Osteogenic differentiation was confirmed by detecting ALP activity using a BCIP/NBT tablet kit (Sigma) and intracytoplasmic calcium crystals were visualized using Alizarin red staining (Lifeline Cell Technology). Chondrogenic differentiation was confirmed with an Alcian blue staining kit (Lifeline Cell Technology).

### Attenuation of Tumor Formation by Levamisole Treatment

All NOD/SCID mice were initially inoculated i.p. with 1 × 10^6^ CP70 cells. Three days after the injection, the mice were divided into two groups that received either 50 mg/kg levamisole (Sigma) or vehicle orally every day for 21 days. To evaluate the levamisole treatment response of tumors, fluorodeoxyglucose (FDG)/micro-positron emission tomography (PET) scanning was performed 4 weeks after tumor inoculation. The mice anesthetized with isoflurane were injected intravenously with 250 µCi ^18^F-FDG and were imaged 30 minutes later using a BioPET system (Bioscan, Washington, DC).

### Tissue Microarray and Immunohistochemistry Analyses

Paraffin-embedded tumor tissues and tissue microarray slides were analyzed by the Department of Pathology, National Defense Medical Center, Taipei, Taiwan [21, 46]. Seventy-three ovarian surface epithelial carcinomas (including 50 serous cystadenocarcinomas and 23 other types of carcinomas) were collected and analyzed in the tissue microarrays. The tissue microarray sections were stained with anti-human ALP polyclonal antibody (GeneTex). To evaluate the histological appearance and ALP immunoreactivity, two pathologists screened the histological sections and selected areas of representative tumor cells for scoring. The methods for obtaining pathology data for each specimen adhered to the protocol approved by the institutional review board.

### Statistical Analysis

The SPSS software package (version 13 for Windows; IBM Corp., Armonk, NY) was used for statistical analysis. Correlations between ALP expression and clinicopathological characteristics were identified by the χ^2^ test or Fisher's exact test. The disease-free interval and overall survival time were assessed by Cox regression analysis. Kaplan–Meier survival curves were compared using the log-rank and Breslow test. The significance level was defined as *p*<.05.

## Supplemental Figures


