# Supplementary material for: Ovarian cancer stem-like cells with induced translineage-differentiation capacity and are suppressed by alkaline phosphatase inhibitor
Source: Oncotarget. 2013 Nov 16;4(12):2366–82. doi: 10.18632/oncotarget.1424 (PMC3926833; doi:10.18632/oncotarget.1424)
Supplement: Supplementary file 1 [file oncotarget-04-2366-s001.ppt]

## Slide 1
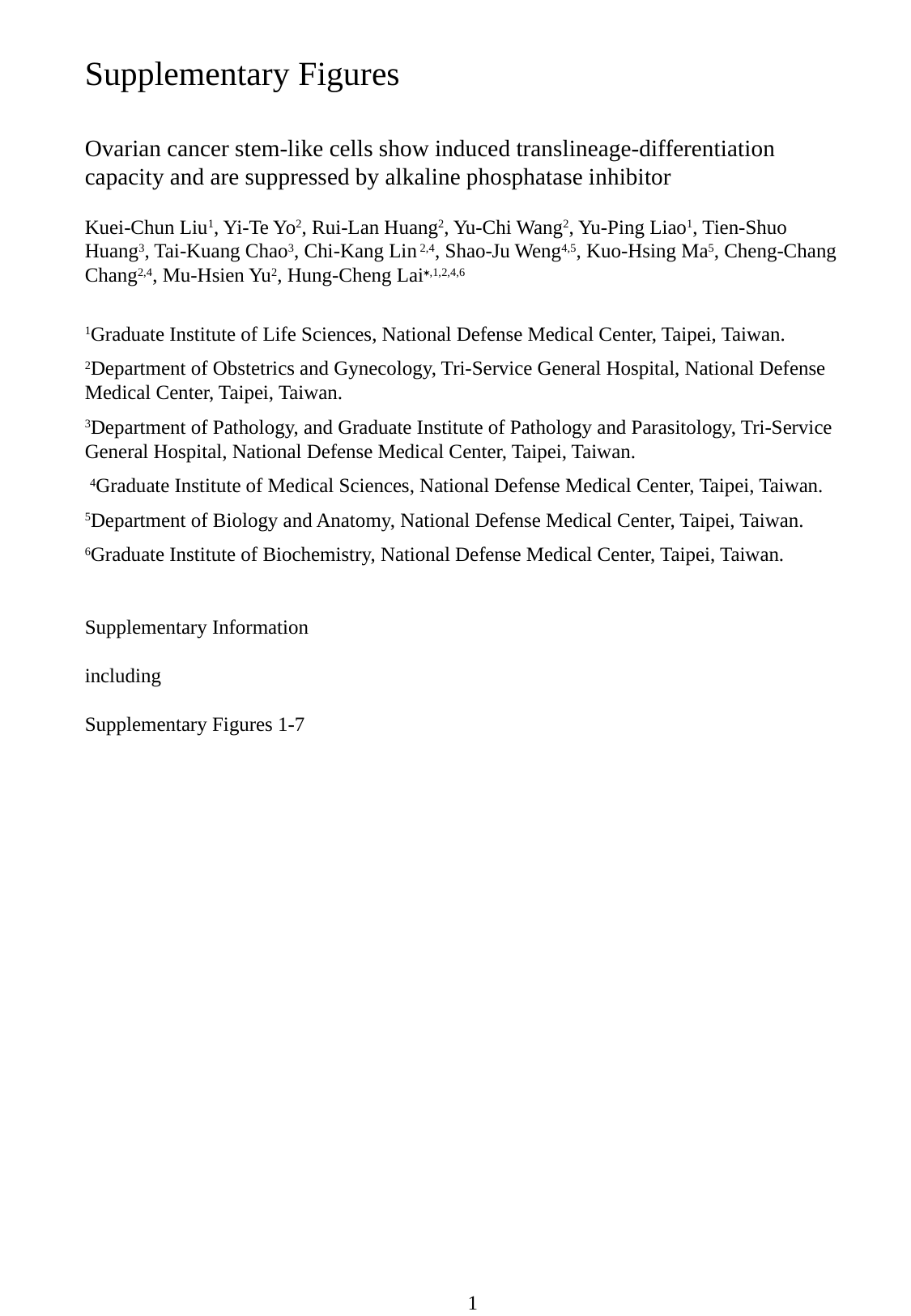

Supplementary Figures
Ovarian cancer stem-like cells show induced translineage-differentiation capacity and are suppressed by alkaline phosphatase inhibitor
Kuei-Chun Liu1, Yi-Te Yo2, Rui-Lan Huang2, Yu-Chi Wang2, Yu-Ping Liao1, Tien-Shuo Huang3, Tai-Kuang Chao3, Chi-Kang Lin 2,4, Shao-Ju Weng4,5, Kuo-Hsing Ma5, Cheng-Chang Chang2,4, Mu-Hsien Yu2, Hung-Cheng Lai,1,2,4,6
1Graduate Institute of Life Sciences, National Defense Medical Center, Taipei, Taiwan.
2Department of Obstetrics and Gynecology, Tri-Service General Hospital, National Defense Medical Center, Taipei, Taiwan.
3Department of Pathology, and Graduate Institute of Pathology and Parasitology, Tri-Service General Hospital, National Defense Medical Center, Taipei, Taiwan.
 4Graduate Institute of Medical Sciences, National Defense Medical Center, Taipei, Taiwan.
5Department of Biology and Anatomy, National Defense Medical Center, Taipei, Taiwan.
6Graduate Institute of Biochemistry, National Defense Medical Center, Taipei, Taiwan.
Supplementary Information
including
Supplementary Figures 1-7
1

## Slide 2
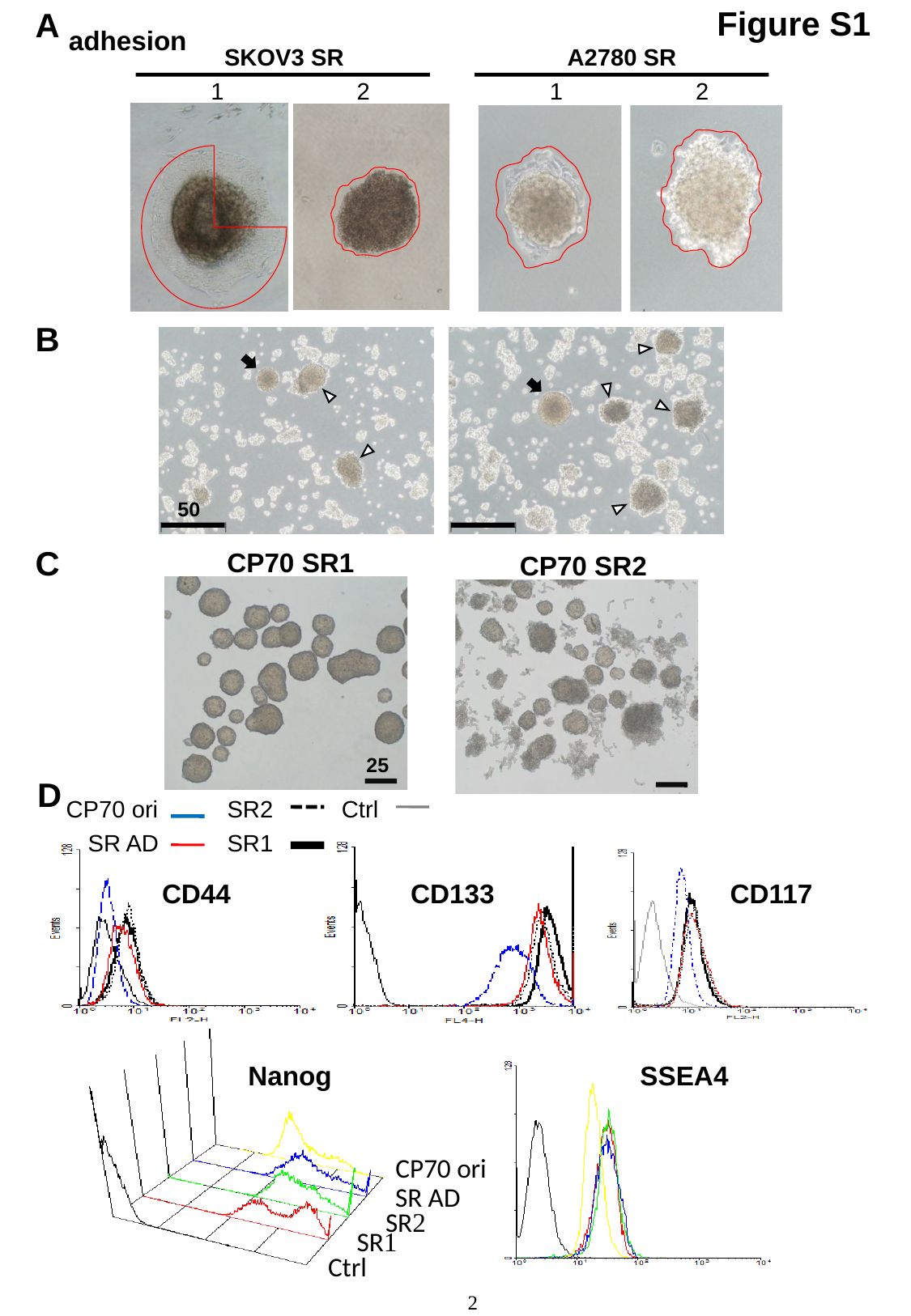

Figure S1
A
adhesion
SKOV3 SR
A2780 SR
1
2
1
2
B
50
C
CP70 SR1
CP70 SR2
25
D
CP70 ori
SR2
Ctrl
SR AD
SR1
CD44
CD133
CD117
Nanog
SSEA4
CP70 ori
SR AD
SR
SR
Ctrl
2

## Slide 3
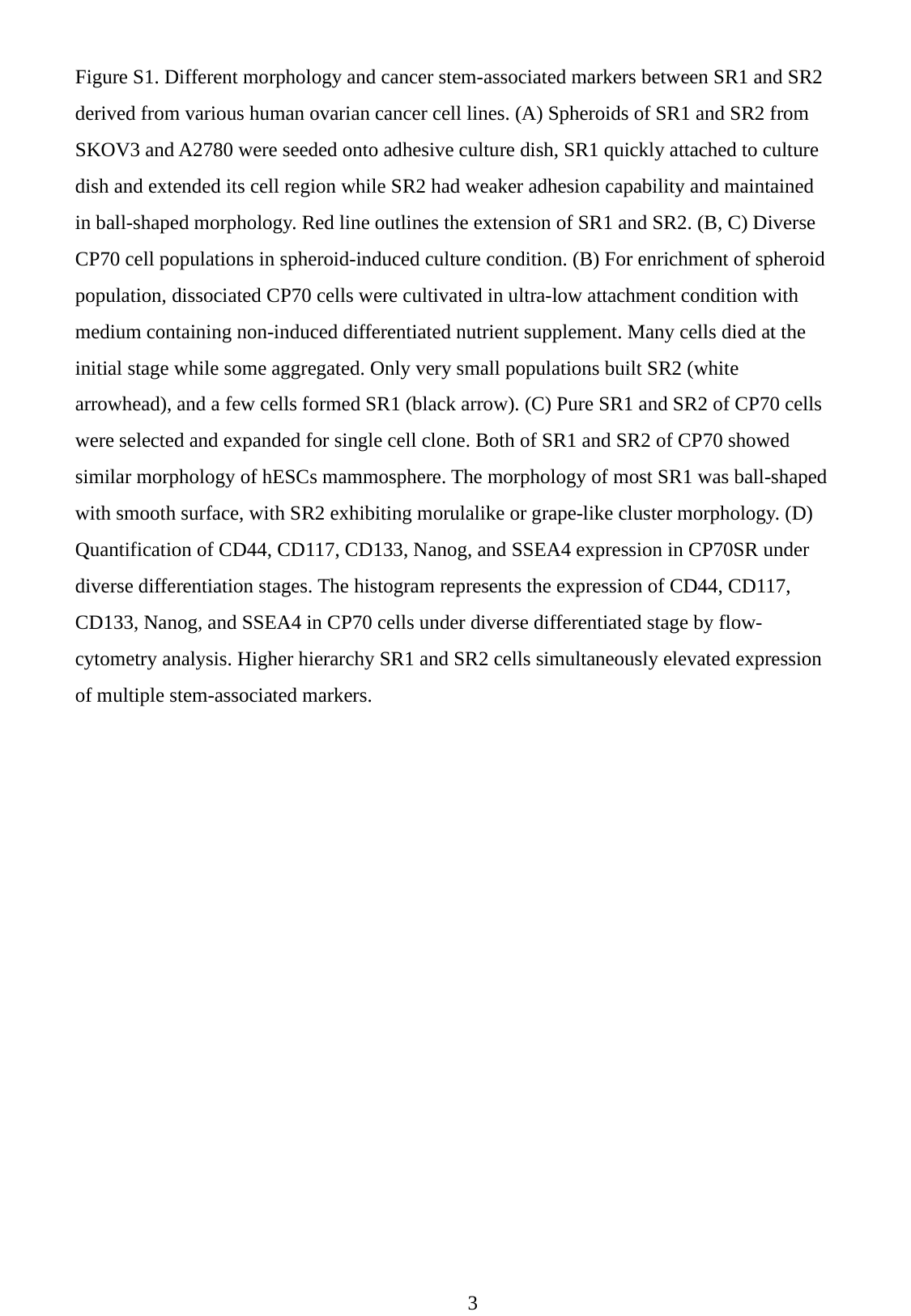

Figure S1. Different morphology and cancer stem-associated markers between SR1 and SR2 derived from various human ovarian cancer cell lines. (A) Spheroids of SR1 and SR2 from SKOV3 and A2780 were seeded onto adhesive culture dish, SR1 quickly attached to culture dish and extended its cell region while SR2 had weaker adhesion capability and maintained in ball-shaped morphology. Red line outlines the extension of SR1 and SR2. (B, C) Diverse CP70 cell populations in spheroid-induced culture condition. (B) For enrichment of spheroid population, dissociated CP70 cells were cultivated in ultra-low attachment condition with medium containing non-induced differentiated nutrient supplement. Many cells died at the initial stage while some aggregated. Only very small populations built SR2 (white arrowhead), and a few cells formed SR1 (black arrow). (C) Pure SR1 and SR2 of CP70 cells were selected and expanded for single cell clone. Both of SR1 and SR2 of CP70 showed similar morphology of hESCs mammosphere. The morphology of most SR1 was ball-shaped with smooth surface, with SR2 exhibiting morulalike or grape-like cluster morphology. (D) Quantification of CD44, CD117, CD133, Nanog, and SSEA4 expression in CP70SR under diverse differentiation stages. The histogram represents the expression of CD44, CD117, CD133, Nanog, and SSEA4 in CP70 cells under diverse differentiated stage by flow-cytometry analysis. Higher hierarchy SR1 and SR2 cells simultaneously elevated expression of multiple stem-associated markers.
3

## Slide 4
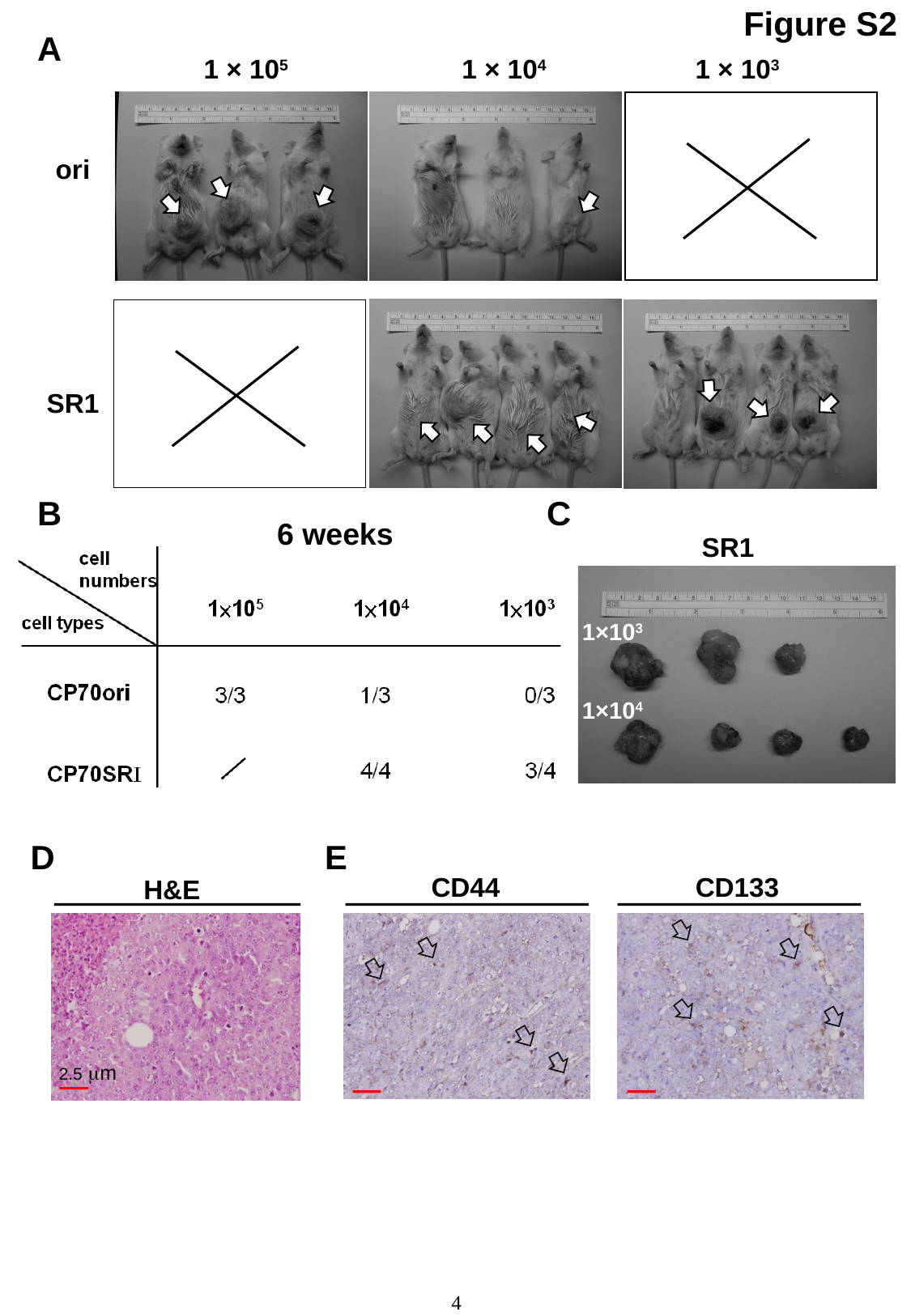

Figure S2
A
1 × 10^5^
1 × 10^4^
1 × 10^3^
ori
SR1
B
C
6 weeks
SR1
1×10^3^
1×10^4^
D
E
CD44
CD133
H&E
2.5 μm
4

## Slide 5
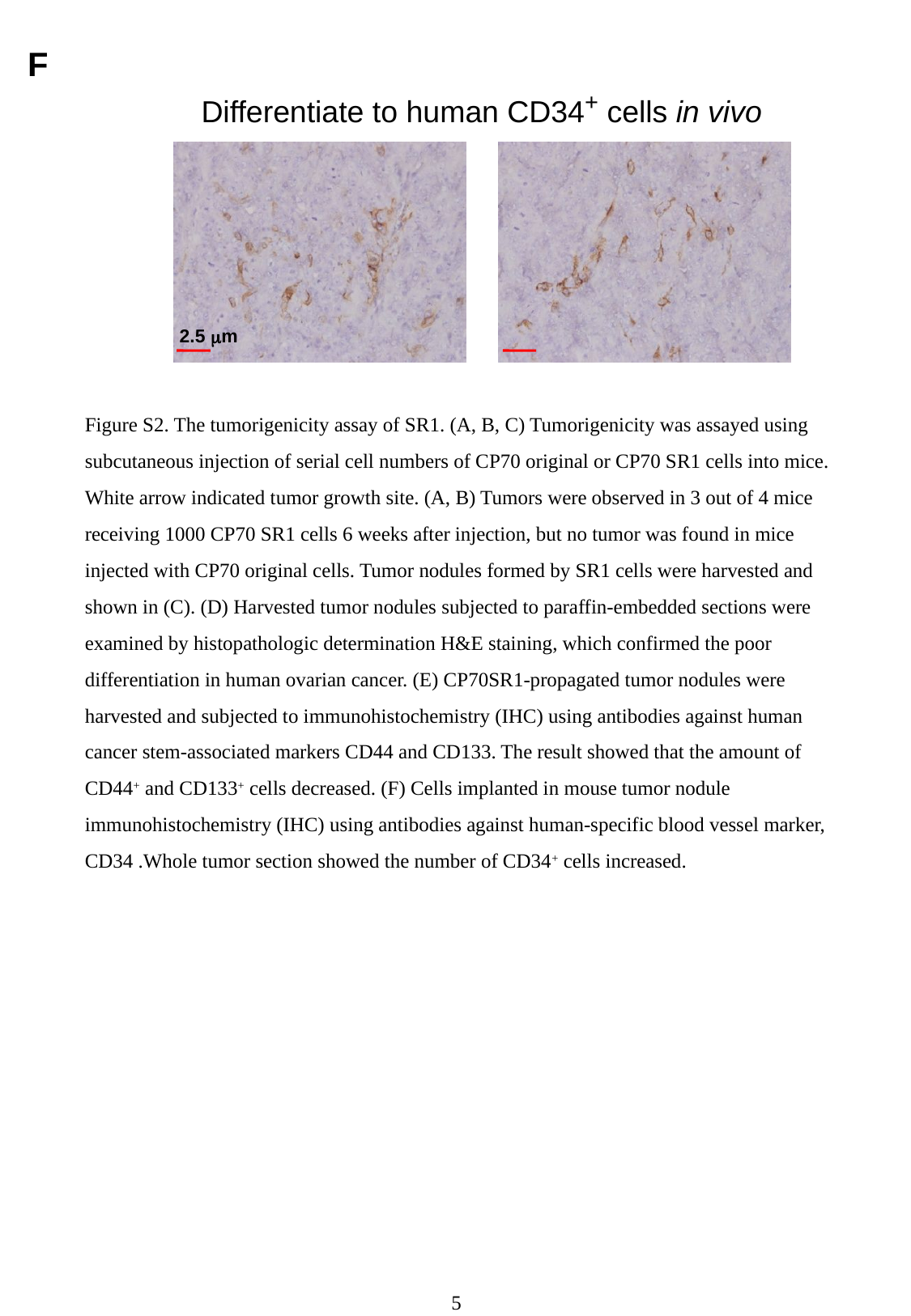

F
Differentiate to human CD34+ cells in vivo
2.5 μm
Figure S2. The tumorigenicity assay of SR1. (A, B, C) Tumorigenicity was assayed using subcutaneous injection of serial cell numbers of CP70 original or CP70 SR1 cells into mice. White arrow indicated tumor growth site. (A, B) Tumors were observed in 3 out of 4 mice receiving 1000 CP70 SR1 cells 6 weeks after injection, but no tumor was found in mice injected with CP70 original cells. Tumor nodules formed by SR1 cells were harvested and shown in (C). (D) Harvested tumor nodules subjected to paraffin-embedded sections were examined by histopathologic determination H&E staining, which confirmed the poor differentiation in human ovarian cancer. (E) CP70SR1-propagated tumor nodules were harvested and subjected to immunohistochemistry (IHC) using antibodies against human cancer stem-associated markers CD44 and CD133. The result showed that the amount of CD44+ and CD133+ cells decreased. (F) Cells implanted in mouse tumor nodule immunohistochemistry (IHC) using antibodies against human-specific blood vessel marker, CD34 .Whole tumor section showed the number of CD34+ cells increased.
5

## Slide 6
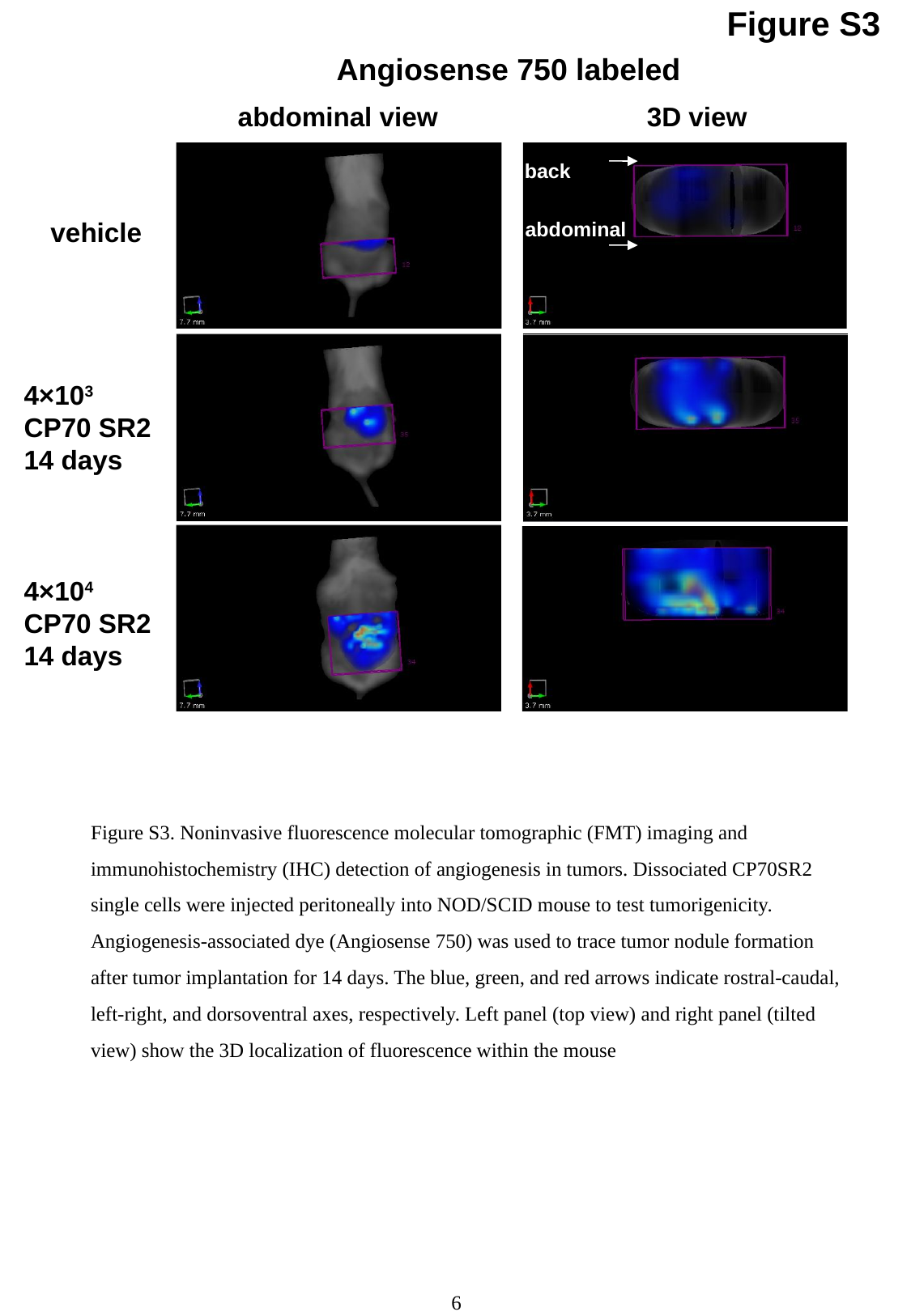

Figure S3
Angiosense 750 labeled
abdominal view
3D view
back
vehicle
abdominal
4×10^3^
CP70 SR2
14 days
4×10^4^
CP70 SR2
14 days
Figure S3. Noninvasive fluorescence molecular tomographic (FMT) imaging and immunohistochemistry (IHC) detection of angiogenesis in tumors. Dissociated CP70SR2 single cells were injected peritoneally into NOD/SCID mouse to test tumorigenicity. Angiogenesis-associated dye (Angiosense 750) was used to trace tumor nodule formation after tumor implantation for 14 days. The blue, green, and red arrows indicate rostral-caudal, left-right, and dorsoventral axes, respectively. Left panel (top view) and right panel (tilted view) show the 3D localization of fluorescence within the mouse
6

## Slide 7
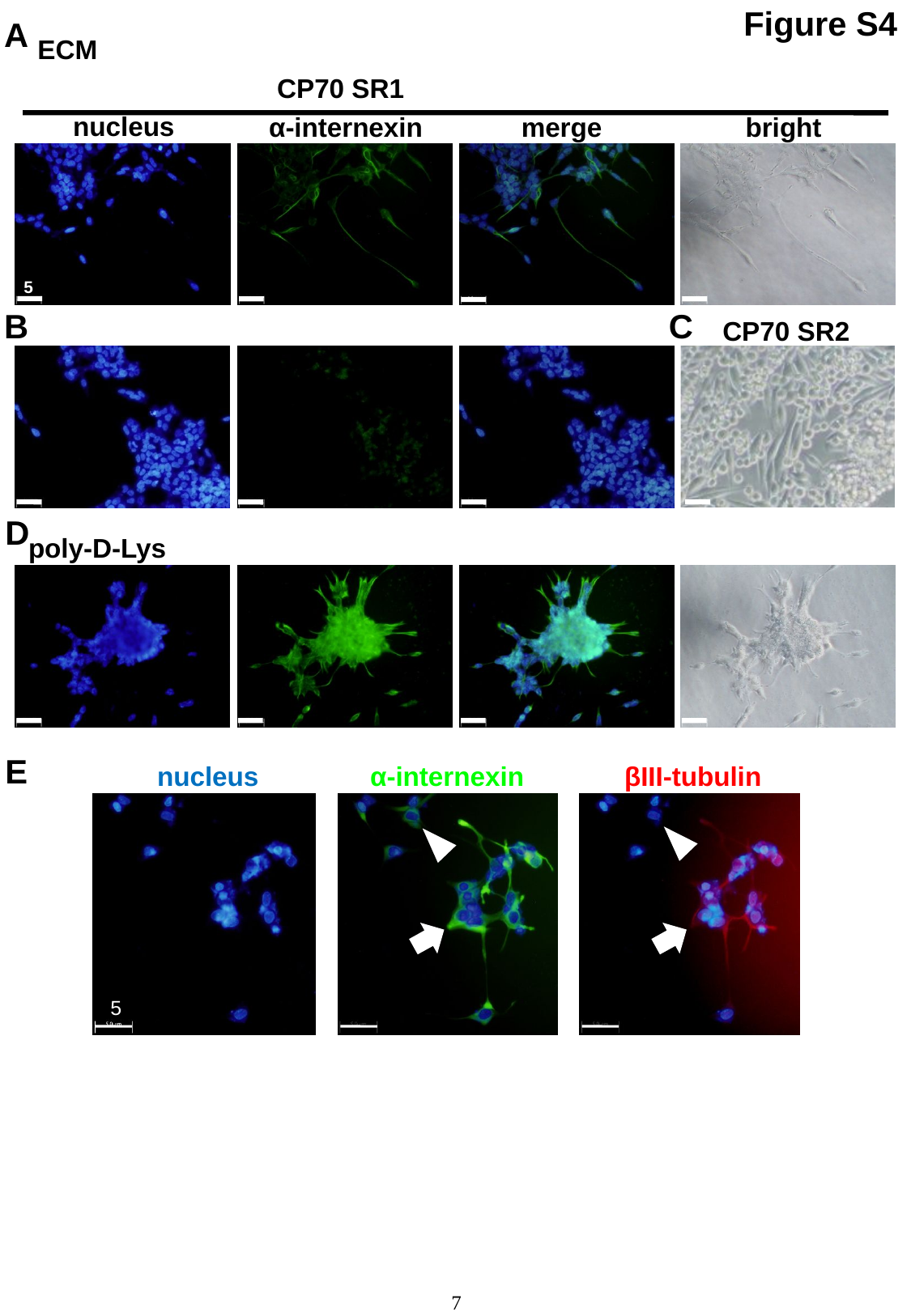

Figure S4
A
ECM
CP70 SR1
nucleus
α-internexin
merge
bright
5
B
C
CP70 SR2
D
poly-D-Lys
E
nucleus
α-internexin
βIII-tubulin
5
7

## Slide 8
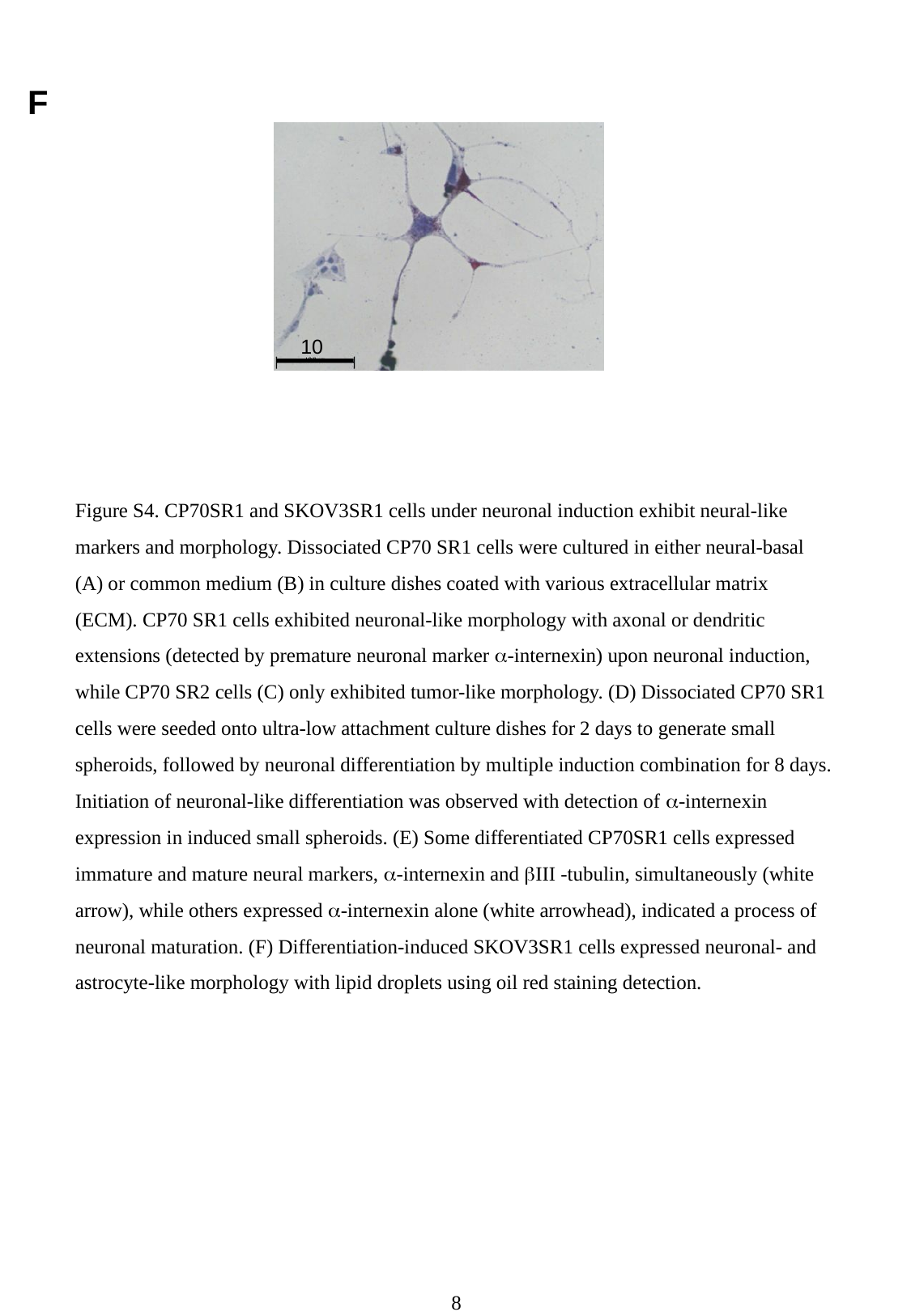

F
10
Figure S4. CP70SR1 and SKOV3SR1 cells under neuronal induction exhibit neural-like markers and morphology. Dissociated CP70 SR1 cells were cultured in either neural-basal (A) or common medium (B) in culture dishes coated with various extracellular matrix (ECM). CP70 SR1 cells exhibited neuronal-like morphology with axonal or dendritic extensions (detected by premature neuronal marker α-internexin) upon neuronal induction, while CP70 SR2 cells (C) only exhibited tumor-like morphology. (D) Dissociated CP70 SR1 cells were seeded onto ultra-low attachment culture dishes for 2 days to generate small spheroids, followed by neuronal differentiation by multiple induction combination for 8 days. Initiation of neuronal-like differentiation was observed with detection of α-internexin expression in induced small spheroids. (E) Some differentiated CP70SR1 cells expressed immature and mature neural markers, α-internexin and βIII -tubulin, simultaneously (white arrow), while others expressed α-internexin alone (white arrowhead), indicated a process of neuronal maturation. (F) Differentiation-induced SKOV3SR1 cells expressed neuronal- and astrocyte-like morphology with lipid droplets using oil red staining detection.
8

## Slide 9
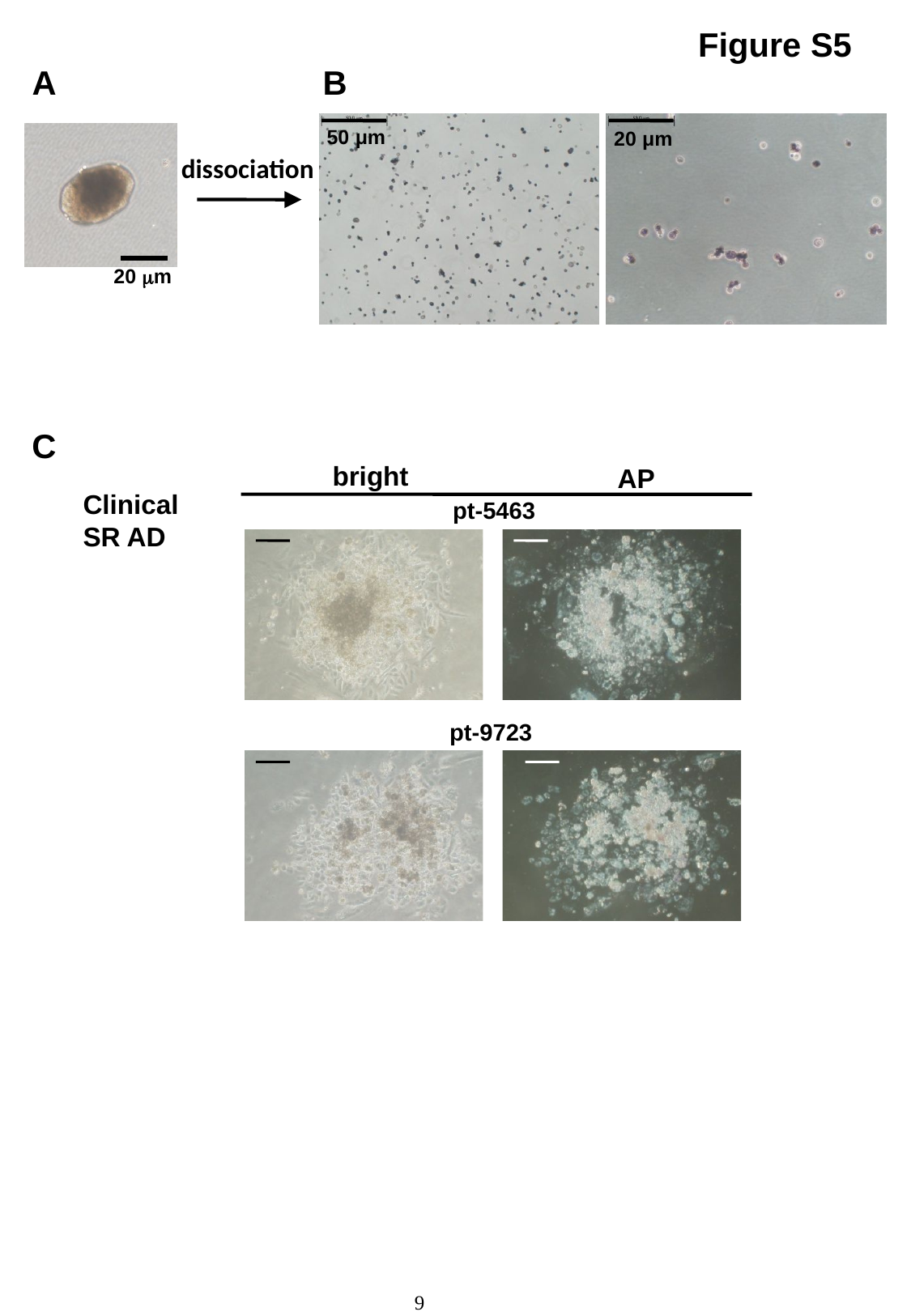

Figure S5
A
B
50 μm
20 μm
dissociation
20 μm
C
bright
AP
pt-5463
pt-9723
Clinical
SR AD
<number>

## Slide 10
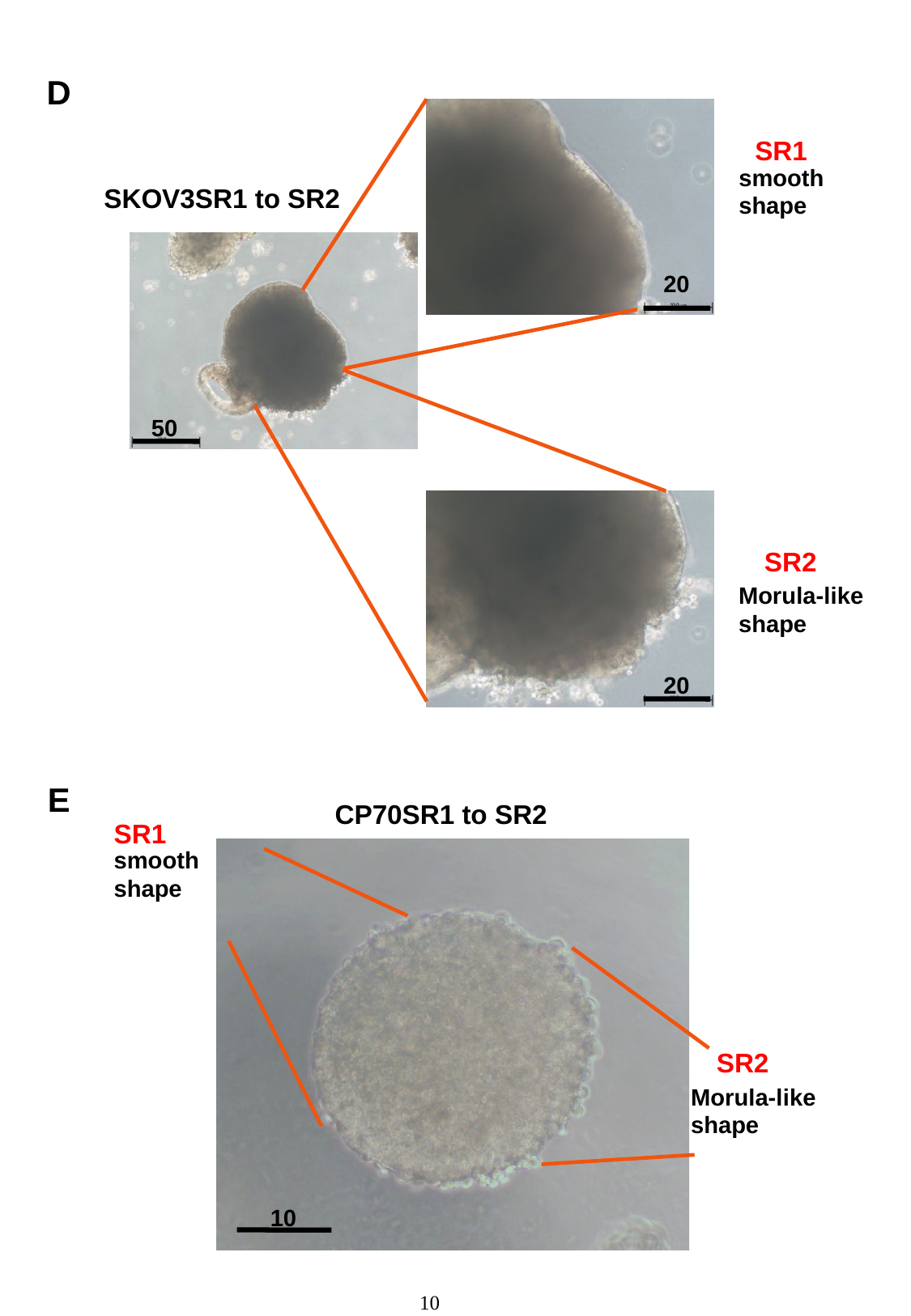

D
SR1
smooth
shape
SKOV3SR1 to SR2
20
50
SR2
Morula-like
shape
20
E
CP70SR1 to SR2
SR1
smooth
shape
SR2
Morula-like
shape
10
<number>

## Slide 11
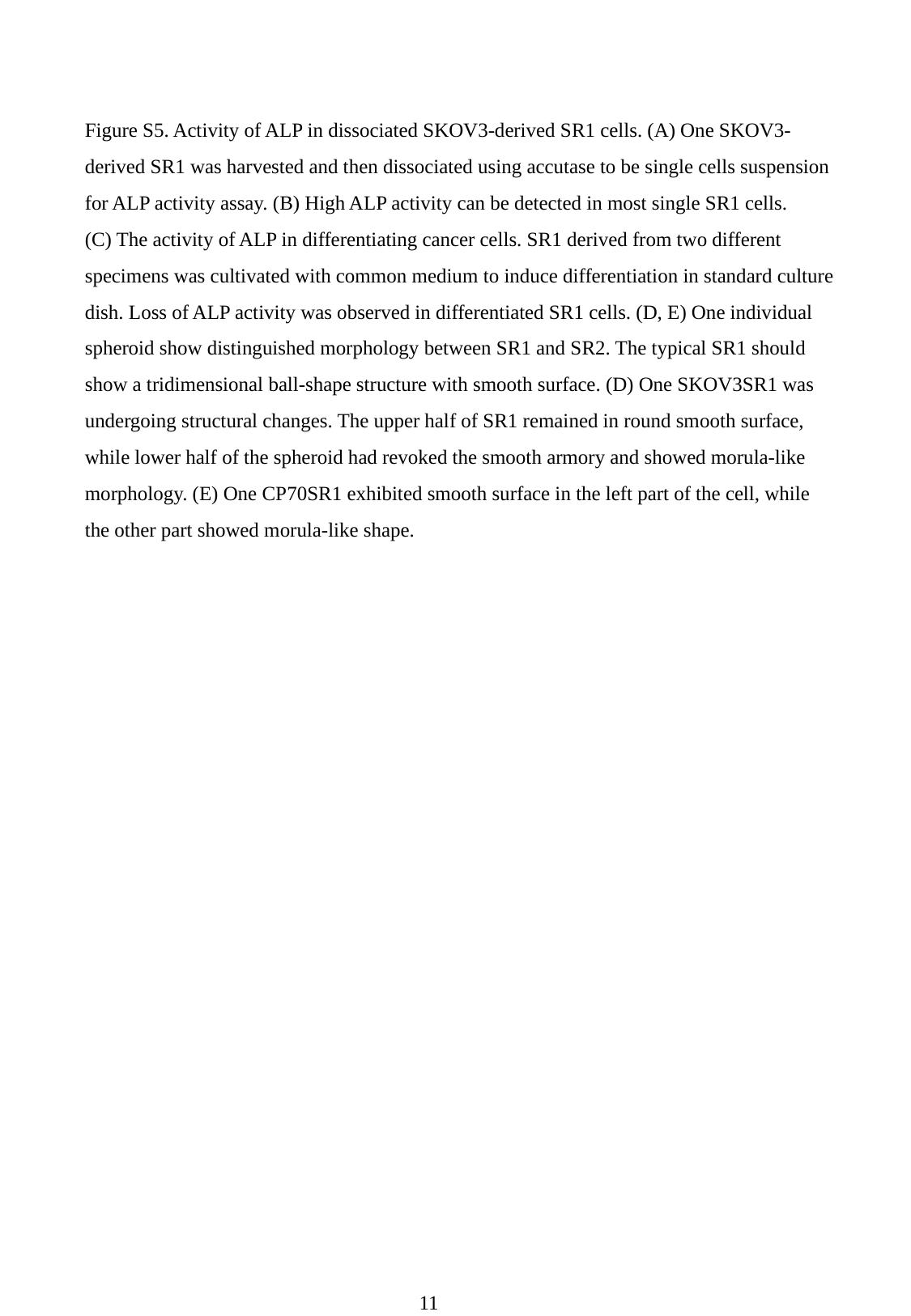

Figure S5. Activity of ALP in dissociated SKOV3-derived SR1 cells. (A) One SKOV3-
derived SR1 was harvested and then dissociated using accutase to be single cells suspension
for ALP activity assay. (B) High ALP activity can be detected in most single SR1 cells.
(C) The activity of ALP in differentiating cancer cells. SR1 derived from two different specimens was cultivated with common medium to induce differentiation in standard culture dish. Loss of ALP activity was observed in differentiated SR1 cells. (D, E) One individual spheroid show distinguished morphology between SR1 and SR2. The typical SR1 should show a tridimensional ball-shape structure with smooth surface. (D) One SKOV3SR1 was undergoing structural changes. The upper half of SR1 remained in round smooth surface, while lower half of the spheroid had revoked the smooth armory and showed morula-like morphology. (E) One CP70SR1 exhibited smooth surface in the left part of the cell, while the other part showed morula-like shape.
<number>

## Slide 12
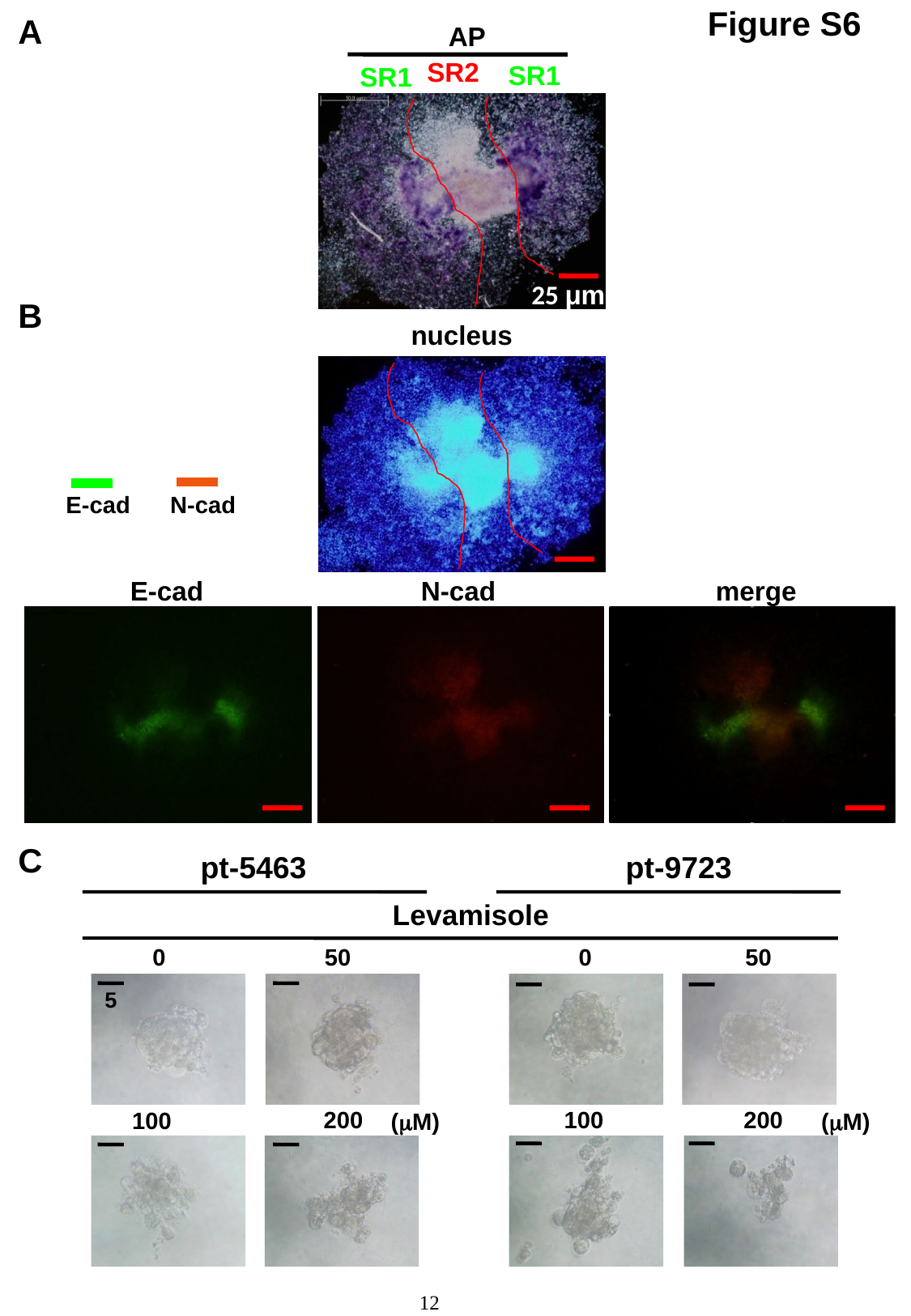

Figure S6
A
AP
SR2
SR1
SR1
25 μm
B
nucleus
E-cad N-cad
E-cad
N-cad
merge
C
pt-5463
pt-9723
Levamisole
0
50
0
50
5
200
100
200
100
(μM)
(μM)
<number>

## Slide 13
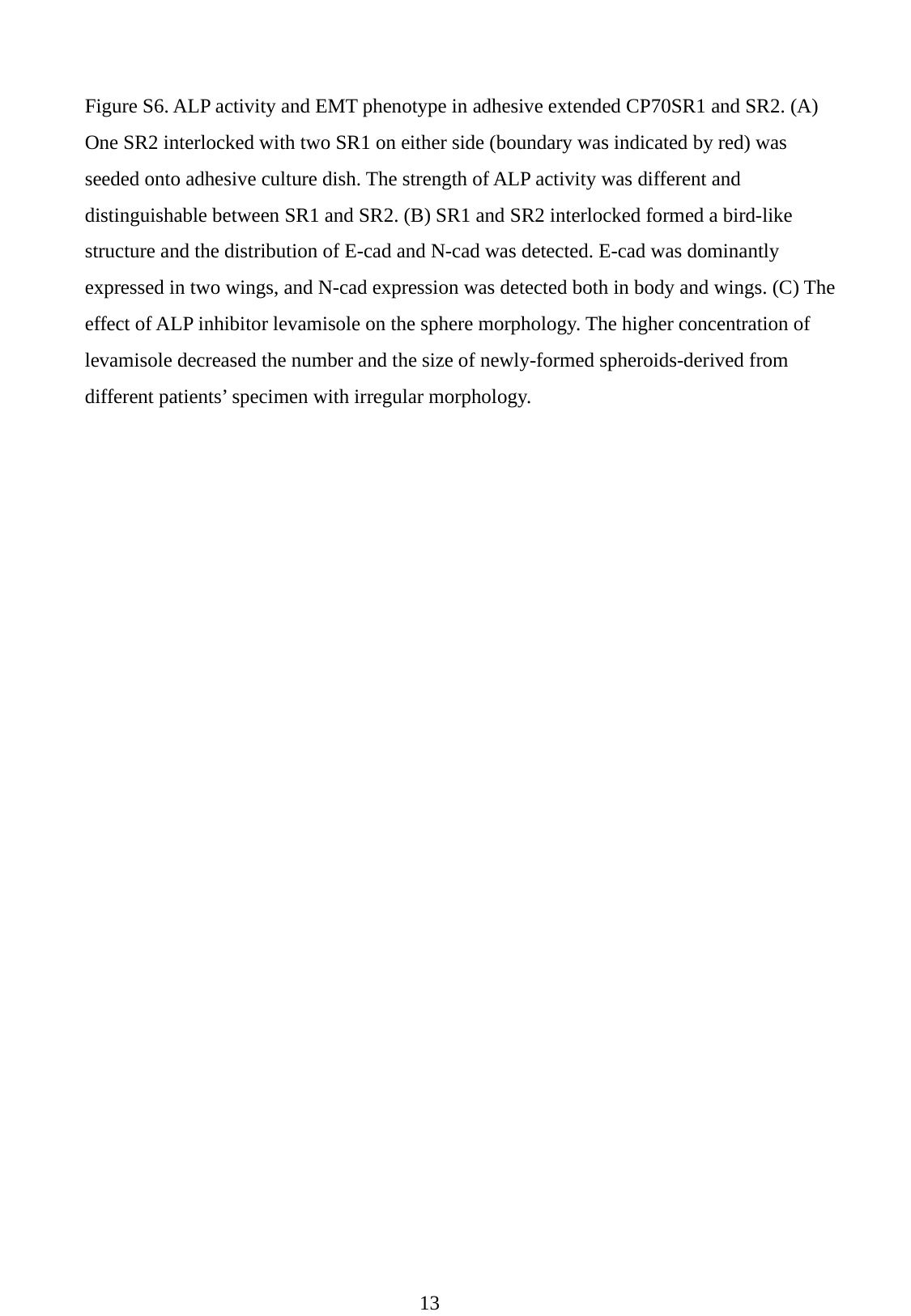

Figure S6. ALP activity and EMT phenotype in adhesive extended CP70SR1 and SR2. (A) One SR2 interlocked with two SR1 on either side (boundary was indicated by red) was seeded onto adhesive culture dish. The strength of ALP activity was different and distinguishable between SR1 and SR2. (B) SR1 and SR2 interlocked formed a bird-like structure and the distribution of E-cad and N-cad was detected. E-cad was dominantly expressed in two wings, and N-cad expression was detected both in body and wings. (C) The effect of ALP inhibitor levamisole on the sphere morphology. The higher concentration of levamisole decreased the number and the size of newly-formed spheroids-derived from different patients’ specimen with irregular morphology.
<number>

## Slide 14
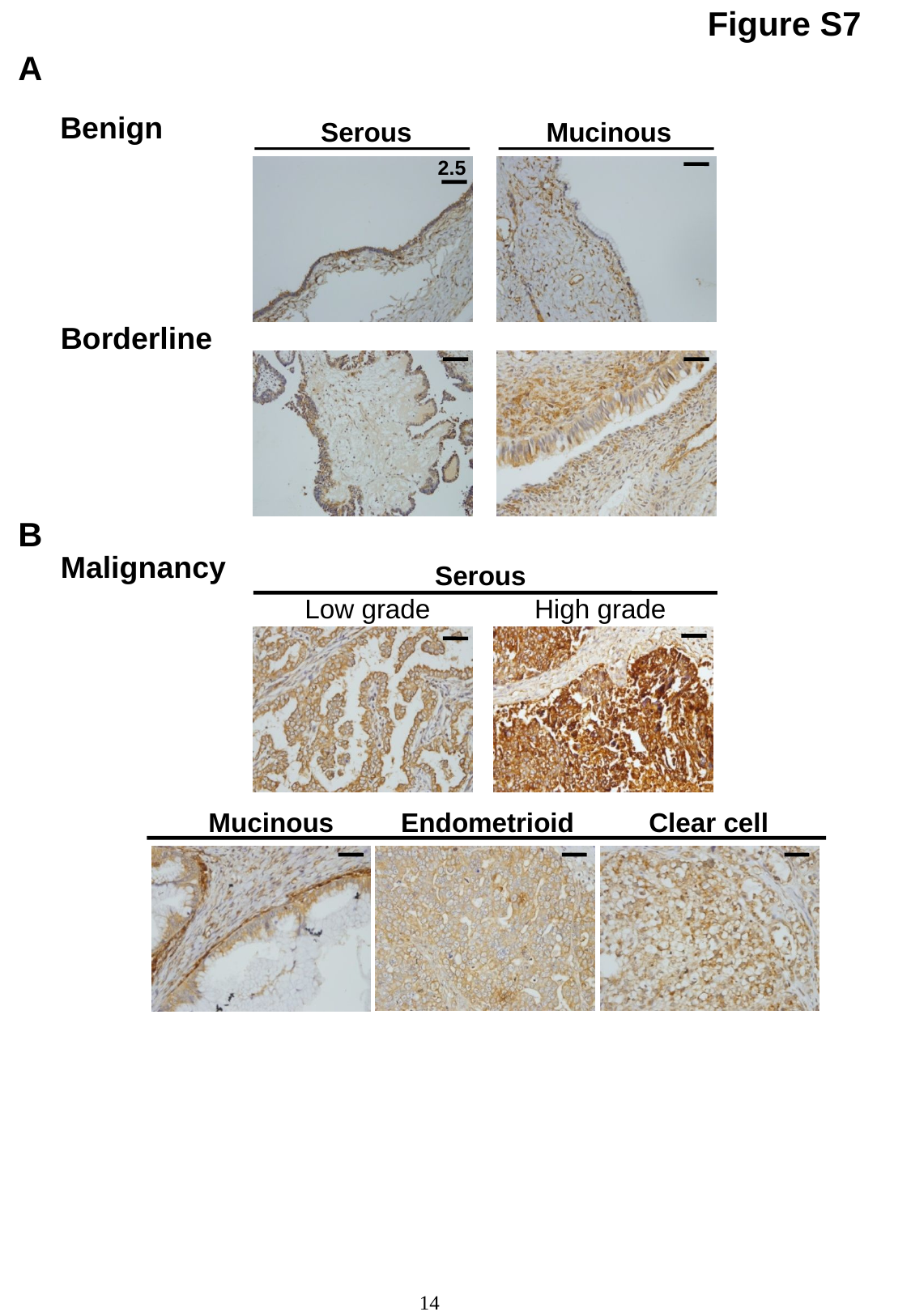

Figure S7
A
Benign
Serous
Mucinous
2.5
Borderline
B
Malignancy
Serous
Low grade
High grade
Mucinous Endometrioid Clear cell
<number>

## Slide 15
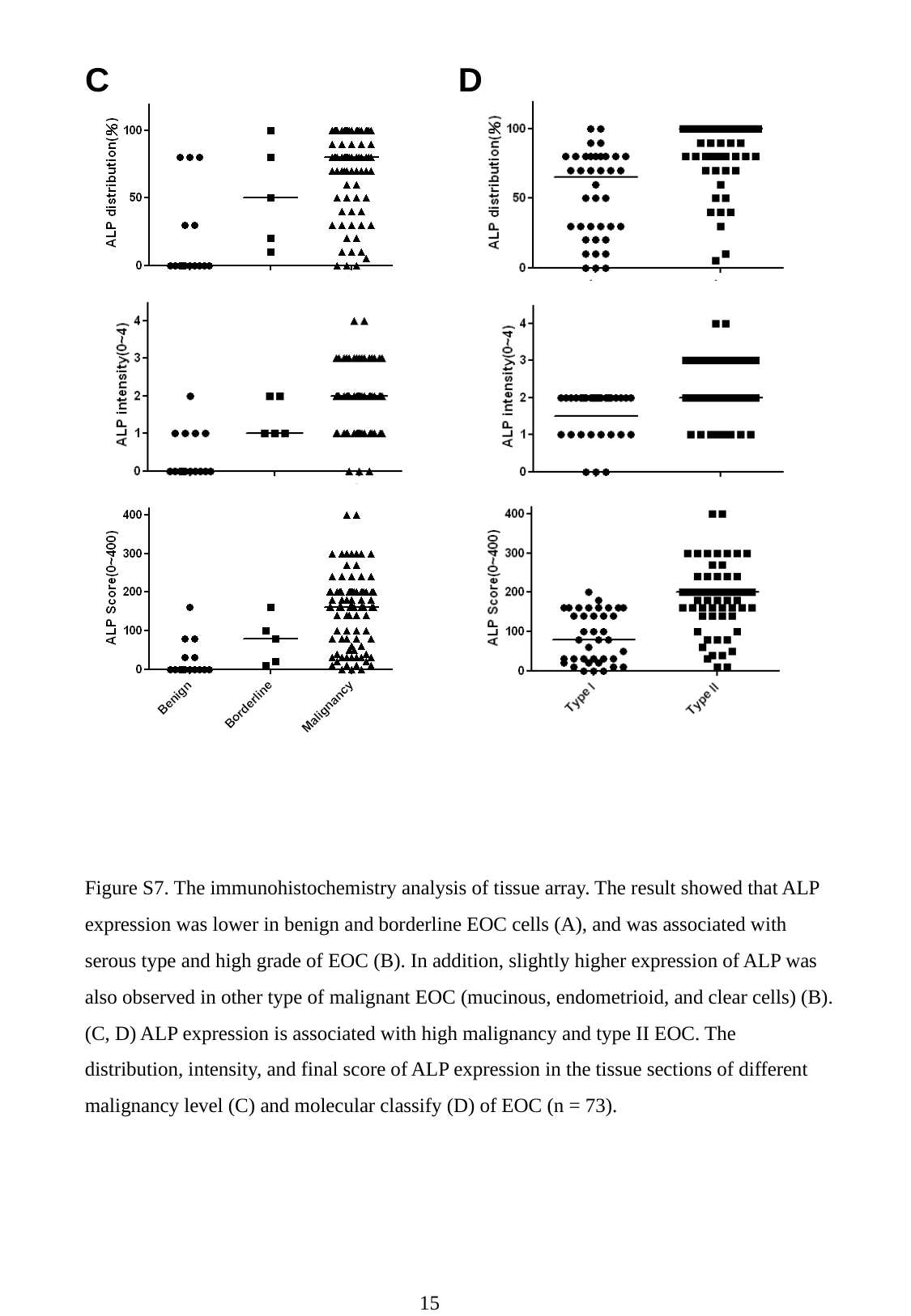

C
D
Figure S7. The immunohistochemistry analysis of tissue array. The result showed that ALP expression was lower in benign and borderline EOC cells (A), and was associated with serous type and high grade of EOC (B). In addition, slightly higher expression of ALP was also observed in other type of malignant EOC (mucinous, endometrioid, and clear cells) (B). (C, D) ALP expression is associated with high malignancy and type II EOC. The distribution, intensity, and final score of ALP expression in the tissue sections of different malignancy level (C) and molecular classify (D) of EOC (n = 73).
<number>
